# Semiconducting polymer nano-PROTACs for activatable photo-immunometabolic cancer therapy

**DOI:** 10.1038/s41467-021-23194-w

**Published:** 2021-05-18

**Authors:** Chi Zhang, Ziling Zeng, Dong Cui, Shasha He, Yuyan Jiang, Jingchao Li, Jiaguo Huang, Kanyi Pu

**Affiliations:** 1 School of Chemical and Biomedical Engineering, Nanyang Technological University, Singapore, Singapore; 2 Division of Chemistry and Biological Chemistry, School of Physical and Mathematical Sciences, Nanyang Technological University, Singapore, Singapore

**Keywords:** Cancer immunotherapy, Nanotechnology in cancer

## Abstract

Immunometabolic intervention has been applied to treat cancer via inhibition of certain enzymes associated with intratumoral metabolism. However, small-molecule inhibitors and genetic modification often suffer from insufficiency and off-target side effects. Proteolysis targeting chimeras (PROTACs) provide an alternative way to modulate protein homeostasis for cancer therapy; however, the always-on bioactivity of existing PROTACs potentially leads to uncontrollable protein degradation at non-target sites, limiting their in vivo therapeutic efficacy. We herein report a semiconducting polymer nano-PROTAC (SPN_pro_) with phototherapeutic and activatable protein degradation abilities for photo-immunometabolic cancer therapy. SPN_pro_ can remotely generate singlet oxygen (^1^O_2_) under NIR photoirradiation to eradicate tumor cells and induce immunogenic cell death (ICD) to enhance tumor immunogenicity. Moreover, the PROTAC function of SPN_pro_ is specifically activated by a cancer biomarker (cathepsin B) to trigger targeted proteolysis of immunosuppressive indoleamine 2,3-dioxygenase (IDO) in the tumor of living mice. The persistent IDO degradation blocks tryptophan (Trp)-catabolism program and promotes the activation of effector T cells. Such a SPNpro-mediated in-situ immunometabolic intervention synergizes immunogenic phototherapy to boost the antitumor T-cell immunity, effectively inhibiting tumor growth and metastasis. Thus, this study provides a polymer platform to advance PROTAC in cancer therapy.

## Abstract (web summary)

Proteolysis targeting chimeras (PROTACs) is an effective alternative to modulate protein homeostasis but can lead to uncontrollable protein degradation and off-target side effects. Here, the authors developed semiconducting polymer nano-PROTACs with phototherapeutic and activatable protein degradation abilities for photo-immunometabolic cancer therapy.

## Introduction

Cancer immunotherapy that leverages the innate and adaptive immune systems to fight against cancer has revolutionized the treatment of malignancies^1^. However, cancer immunotherapy often has low patient response rate and high risk of immune-related adverse events (irAEs, e.g., endocrinopathy, pneumonitis, hepatitis, nephritis, colitis, etc.), which greatly restrict the clinical applications^2^. Nanomedicines have shown promise to mitigate both issues in preclinical settings owing to their ability to modulate the systemic biodistribution and targeted accumulation of administered immunotherapeutic agents^3^. In particular, activatable immunotherapeutic nanoagents that modulate their immunotherapeutic action in response to cancer biomarkers have been developed to further improve the therapeutic specificity for immune checkpoint inhibitors, immunomodulatory drugs, antibodies, and adjuvants^4–6^. For example, a pH-sensitive dextran nanoparticle was developed to selectively activate anti-PD-1 antibody in mildly acidic tumor microenvironment (TME) for checkpoint blockade immunotherapy^7^, and a cell surface-conjugated protein nanogel was designed to selectively activate the antitumor immunity of T cells in response to the increased reduction potential on the surface of T cells^8^. These studies clearly validated the feasibility of activatable immunotherapeutic nanoagents to increase therapeutic efficacy and reduce irAEs.

Immunometabolism that involves a network of intracellular metabolic pathways such as glycolysis, the tricarboxylic acid cycle, the pentose phosphate pathway, and amino acid metabolism plays a crucial role in modulating the responses of immune cells^9^. Particularly, the metabolism of amino acids such as tryptophan (Trp), arginine, glutamine, and leucine can affect both tumor progression and proliferation and differentiation of immune cells^10^. Thus, immunometabolic cancer therapy has been developed based on the inhibition of rate-limiting enzymes associated with the metabolism of these amino acids^11^. However, small-molecule inhibitors are generally unable to afford durable response due to the existence of undruggable proteins and drug resistance^12^, while genetic methods often suffer from insufficient transfection efficiency and off-target side effects^13^. Thus, alternative methods are highly desired to intervene in the metabolism of amino acids for cancer immunotherapy.

PROTAC (proteolysis targeting chimera), which comprises two covalently linked moieties to bind protein of interest (POI) and E3 ligase, respectively, has recently emerged as a powerful tool for targeted posttranslational knockdown of POI^14,15^. The heterobifunctional structure of PROTAC allows dictating the POI for proteolysis via the ubiquitin-proteasome system^16^. As PROTAC-induced protein degradation is a repetitional process, it can potentially have more persistent action than inhibitors or genetic tools, consequently lowering the administration doses. Till now, PROTACs have been applied to modulate the level of essential proteins including the bromodomain and extra-terminal (BET) family proteins BRD2-4^17^, signal transducer and activator of transcription (STAT) family protein STAT3^18^, and cyclin-dependent kinases 4 and 6 for cancer therapy^19^. However, PROTACs have been rarely developed to modulate the proteolysis of immunometabolism-associated proteins for cancer immunometabolic therapy; moreover, no smart activatable PROTACs have been developed to minimize the issues of always-on bioactivity and off-target side effects.

**Fig. 1:**
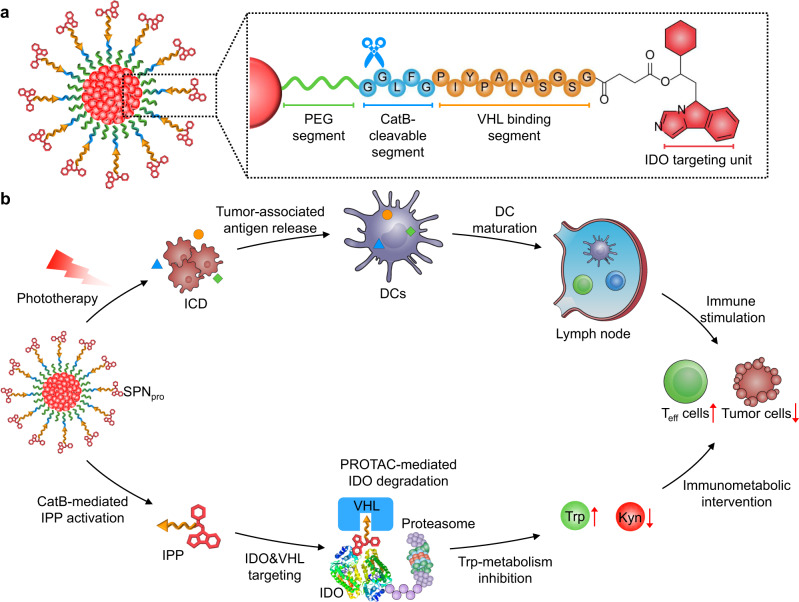
Schematic illustration of SPN_pro_-mediated IDO degradation for cancer photo-immunometabolic therapy. **a** Structure and cathepsin B (CatB)-specific activation mechanism of SPN_pro_. **b** SPN_pro_-mediated activatable photo-immunometabolic therapy with two processes: (i) a series of cancer immune responses including immunogenic cell death (ICD), tumor-associated antigen release, DC maturation, and effector T (T_eff_) cell activation upon NIR photoirradiation; (ii) SPN_pro_-mediated immunometabolic intervention processes including CatB-specific activation of IPP, IDO and VHL targeting, proteasome recruitment, IDO degradation, Trp upregulation and Kyn depletion, and T_eff_ cell activation.

**Fig. 2:**
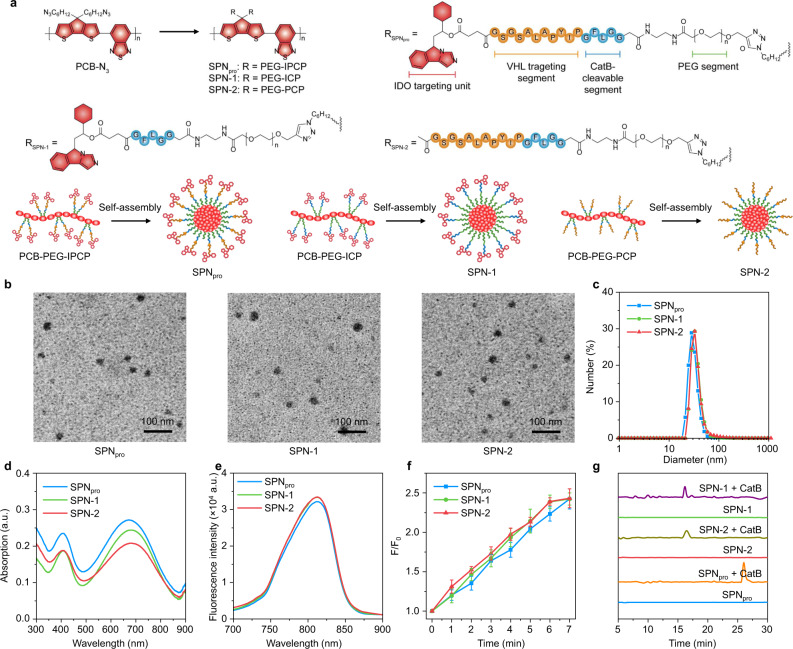
Synthesis and characterization of SPN_pro_, SPN-1, and SPN-2. **a** The molecular structures and syntheses of SPN_pro_, SPN-1, and SPN-2. **b** TEM images of SPN_pro_ (left), SPN-1 (middle), and SPN-2 (right). **c** DLS profiles of SPN_pro_, SPN-1, and SPN-2 in 1× PBS buffer (pH 7.4). UV/Vis absorption (**d**) and fluorescence spectra (**e**) of SPN_pro_, SPN-1, and SPN-2 in 1× PBS buffer (pH 7.4) with an excitation wavelength at 650 nm. **f** The generation of ^1^O_2_ in SPN_pro_, SPN-1, and SPN-2 in 1× PBS buffer (pH 7.4) ([PCB] = 20 μg/mL) as a function of photoirradiation time. The mean values and SD are presented. Error bars represent the standard deviation of three separate measurements (*n* = 3). **g** HPLC profiles of SPN_pro_, SPN-1, and SPN-2 ([PCB] = 20 μg/mL) in the absence or presence of CatB (0.2 U/mL). The experiments in **b** were repeated independently three times with similar results.

We herein report the development of a semiconducting polymer nano-PROTAC (SPN_pro_) for cancer-activated photo-immunometabolic therapy (Fig. 1). SPN_pro_ is composed of a semiconducting polymer core conjugated with PROTAC segments via cancer-biomarker-cleavable peptide (Fig. 2a). Semiconducting polymer nanoparticles (SPNs) have good biocompatibility and tunable optical properties^20–22^, and thus are chosen as phototheranostic components. Indoleamine 2,3-dioxygenase (IDO) is selected as the POI for PROTAC, because it is a Trp-catabolizing enzyme overexpressed in tumor tissues that converts Trp to kynurenine (Kyn), leading to the dysfunction of dendritic cells (DCs) and the suppression of effector T cells^23^. The IDO-targeting PROTAC peptide (IPP) is made of an IDO-targeting unit (a widely used IDO inhibitor, NLG919) and an E3 ubiquitin ligase VHL (the von Hippel Lindau protein)-binding peptide^24,25^. Cathepsin B (CatB) is chosen as the cancer biomarker, which is commonly overexpressed in various cancer cells, such as breast cancer, colorectal cancer, melanoma, and prostate cancer^26^.

The mechanism of SPN_pro_-mediated activatable photo-immunometabolic cancer therapy is proposed as follows. After systemic administration, SPN_pro_ passively accumulates in the tumor of living mice; it generates singlet oxygen (^1^O_2_) to eliminate tumor cells and induces tumor-associated antigens release and immunogenic cell death (ICD) under NIR photoirradiation. These released tumor-associated antigens further induce DC maturation and promote T-cell activation, facilitating antitumor T-cell immune response. Meanwhile, the tumor-overexpressed CatB cleaves SPN_pro_ and releases IPP in situ. The activated IPP binds to the immunosuppressive IDO and brings it to the E3 ubiquitin ligase VHL ligand, leading to the persistent action on IDO degradation via the ubiquitin-proteasome system. The IDO degradation relieves Trp overconsumption and Kyn accumulation, leading to the reversion of immune suppression. As a result, SPN_pro_-mediated activatable photo-immunometabolic therapy exerts a synergetic action to effectively suppress the progression of tumor in mouse model.

## Results

### Synthesis and characterization

SPN_pro_ was self-assembled from an amphiphilic semiconducting polymer. First, the carbonylated NLG919 was prepared by conjugating the hydroxyl group of NLG919 with succinic anhydride and characterized by ESI-MS (Supplementary Fig. 1) and ^1^H NMR (Supplementary Fig. 2). Then IPP with CatB-cleavable segment (IPCP) was prepared via the standard fluorenylmethyloxycarbonyl (Fmoc) solid-phase peptide synthesis (SPPS) method (Supplementary Fig. 3) and conjugated with the carbonylated NLG919 (Supplementary Figs. 4, 5)^27^. IPCP was further reacted with the poly(ethylene glycol) (PEG) chain (M_w_ = 2000) with one terminal of alkynyl group and the other terminal of amine group via an amide condensation to obtain PEG-IPCP conjugate. ^1^H NMR spectrum of PEG-IPCP exhibited that the coupled efficiency of IPCP to PEG chain was about 80%. Then, an NIR-absorbing semiconducting polymer (poly(cyclopentadithiophene-alt-benzothiadiazole), PCB) with the azide groups (PCB-N_3_) was prepared through the Suzuki polycondensation reaction and further conjugated with PEG-IPCP and mPEG-alkyne (M_w_ = 1000) at a molar ratio of 1:4 to afford the final polymer PCB-PEG-IPCP^28^. Owing to the hydrophilic PEG brushes and hydrophobic PCB backbone, PCB-PEG-IPCP could self-assemble into SPN_pro_ in aqueous solutions. For comparison, two control nanoparticles (SPN-1 and SPN-2) were also prepared with the similar method through the self-assembly of the polymer PCB-PEG-ICP and PCB-PEG-PCP, respectively. All the intermediates (ICP, PCP, PEG-ICP, PEG-PCP, PCB-PEG-ICP, and PCB-PEG-PCP) were characterized by ESI-MS and ^1^H NMR (Supplementary Figs. 6–9).

The physical and optical properties of the nanoparticles were studied. The transmission electron microscopy (TEM) images exhibited a spherical morphology and a uniform size distribution of SPN_pro_, SPN-1, and SPN-2 (Fig. 2b). The dynamic light scattering (DLS) analysis (Fig. 2c and Supplementary Fig. 10) further validated the similar hydrodynamic sizes of SPN_pro_ (28 nm), SPN-1 (32 nm), and SPN-2 (32 nm) and their good stability both in PBS and 10% fetal bovine serum (FBS) solution. SPN_pro_, SPN-1, and SPN-2 had similar UV–vis absorption spectra with two characteristic absorption peaks from the PCB core at 405 and 680 nm and similar fluorescence emission maximum at 820 nm (Fig. 2d, e). These data validated that the physical and optical properties of SPNs did not change with the surface conjugation of various segments.

Afterwards, the photodynamic properties of SPN_pro_, SPN-1, and SPN-2 were studied. The generation of ^1^O_2_ for these nanoparticles under NIR photoirradiation was detected using singlet oxygen sensor green (SOSG) as a fluorescence indicator. The fluorescence intensity of SOSG at 520 nm exhibited a gradual increment for these nanoparticle solutions under NIR photoirradiation (0.3 W/cm^2^ at 808 nm, the maximum permissible exposure for skin), proving the generation of ^1^O_2_ (Fig. 2f). After photoirradiation for 7 min, the SOSG fluorescence intensity of SPN_pro_, SPN-1, and SPN-2 similarly increased by about 2.4-fold. These data indicated that the conjugation of different chimeric peptides (IPCP, PCP, and ICP) did not affect the photodynamic properties of PCB core in SPN_pro_, SPN-1, and SPN-2.

**Fig. 3:**
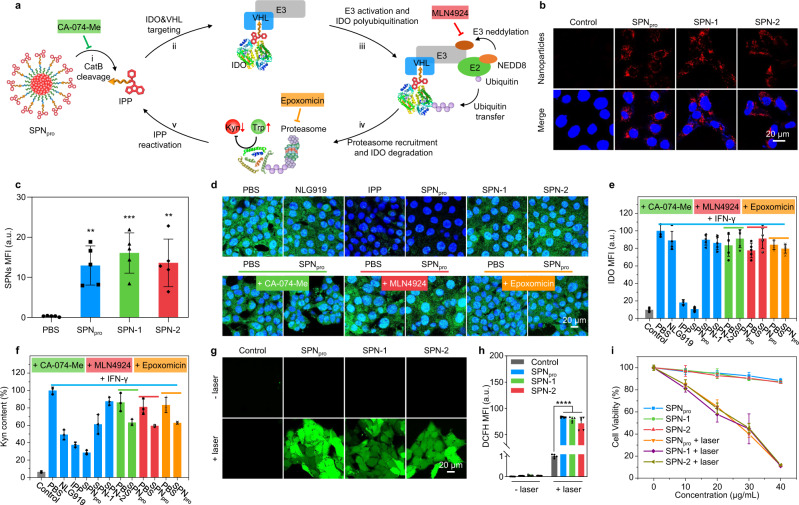
In vitro SPN_pro_-mediated activatable photo-immunometabolic therapy. **a** Proposed mechanism for CatB-specific activation of PROTAC-mediated IDO degradation from SPN_pro_. Confocal fluorescence images (**b**) and MFI (**c**) of 4T1 cancer cells after 24 h incubation with SPN_pro_, SPN-1, and SPN-2 ([PCB] = 20 μg/mL) (*n* = 3). Control versus SPN_pro_: *p* = 0.0025; Control versus SPN-1: *p* = 0.0003; Control versus SPN-2: *p* = 0.0016. Blue fluorescence indicated the cell nucleus stained with 4’,6-diamidino-2-phenylindole (DAPI), and red fluorescence showed the signals from the PCB core. Confocal fluorescence images (**d**) and MFI (**e**) of IFN-γ-stimulated 4T1 cells treated with various inhibitors (CA-074-Me, the CatB inhibitor; MLN4924, the neddylation inhibitor; epoxomicin, the proteasome inhibitor) after 12 h incubation with SPN_pro_, SPN-1, or SPN-2 ([PCB] = 20 μg/mL), followed by staining with green fluorescent anti-IDO antibody (*n* = 3). The cell nucleus was stained with DAPI (blue). **f** Relative Kyn content in the cell culture medium after 12 h incubation with SPN_pro_, SPN-1, or SPN-2 ([PCB] = 20 μg/mL) by HPLC assay (*n* = 3). Confocal fluorescence images (**g**) and MFI (**h**) of 4T1 cells after 12 h incubation with SPN_pro_, SPN-1, or SPN-2 ([PCB] = 20 μg/mL), followed by staining with H_2_DCFDA with or without NIR photoirradiation (0.3 W/cm^2^ at 808 nm) for 6 min (*n* = 3). Control + laser versus SPN_pro_ + laser: *p* < 0.0001; Control + laser versus SPN-1  laser: *p* < 0.0001; Control + laser versus SPN-2 + laser: *p* < 0.0001. Green fluorescence indicated the signals from DCF. **i** Relative cell viabilities of 4T1 cells after 12 h incubation with SPN_pro_, SPN-1, or SPN-2 at different PCB concentrations with or without NIR photoirradiation (0.3 W/cm^2^ at 808 nm) for 6 min (*n* = 3). Statistical significance in **c** and **h** was calculated via one-way ANOVA with a Tukey post-hoc test. **p < 0.0021, ***p < 0.0002, and ****p < 0.0001. The mean values and SD are presented. The experiments in **b** and **d** were repeated independently three times with similar results.

To confirm the CatB-specific activation of SPNs, high-performance liquid chromatography (HPLC) analysis was conducted (Fig. 2g) after incubation of the nanoparticle solutions with CatB. The elution peaks at 26.0, 16.2, and 16.5 min were observed for SPN_pro_, SPN-1, and SPN-2, respectively, which indicated the release of the corresponding chimeric peptides (IPP for SPN_pro_, IDO-targeting peptide (IP) for SPN-1, and PROTAC peptide (PP) for SPN-2) from these nanoparticles (Fig. 3a). In contrast, no such peaks were detected in the absence of CatB, further confirming the CatB-specific activation of SPNs.

### In vitro studies of photo-immunometabolic therapy

To evaluate the cellular uptake of SPNs, 4T1 cancer cells were incubated with SPN_pro_, SPN-1, or SPN-2 for 24 h and then imaged by confocal fluorescence microscopy. The obvious red fluorescence signals from the PCB core could be detected in all SPN-incubated 4T1 cells (Fig. 3b). The relative mean fluorescence intensities (MFIs) of SPN_pro_-, SPN-1-, and SPN-2-incubated 4T1 cells were 13.0, 16.1, and 13.6, respectively, demonstrating their similar cellular uptake by 4T1 cells (Fig. 3c). Meanwhile, the intracellular lysosome colocalization analysis exhibited little overlap between the fluorescence signals of the lysosome and SPNs, which indicated the effective endosomal escape of SPNs after the cellular internalization (Supplementary Fig. 11).

To evaluate the SPN_pro_-mediated IDO degradation, the intracellular IDO expression and Kyn content were detected in 4T1 cells using immunofluorescence staining and HPLC analysis, respectively. 4T1 cells were known to elevate the IDO expression after stimulation with interferon γ (IFN-γ)^29^, which was also confirmed by elevated MFI of FITC-labeled anti-IDO antibodies (10.1-fold) (Fig. 3d, e) and increased Kyn content (15.4-fold) (Fig. 3f) of IFN-γ-stimulated cells relative to the untreated control cells. After incubation of IFN-γ-stimulated cells with IPP or SPN_pro_, the MFIs of FITC-labeled anti-IDO antibodies were greatly decreased by 81.8 and 89.3% relative to that for IFN-γ-stimulated cells, respectively. However, incubation of IFN-γ-stimulated cells with NLG919, SPN-1, or SPN-2 did not decrease the green fluorescence signals of FITC-labeled anti-IDO antibodies. This confirmed that the degradation of IDO was induced by the presence of IPP in SPN_pro_. Meanwhile, incubation of IFN-γ-stimulated cells with IPP or SPN_pro_ led to 62.5 and 71.3% decreases in the relative Kyn content in cells, respectively. Although the decreases of Kyn contents after incubation of IFN-γ-stimulated cells with NLG919 and SPN-1 were observed owing to the IDO inhibition of the NLG919 unit, they only had moderate decreases (50.7% and 38.8%, respectively). These data thus not only validated that SPN_pro_ effectively degraded IDO, but also revealed that the presence of PROTAC unit augmented the decrease of Kyn contents.

The mechanism of SPN_pro_-mediated IDO degradation was further studied. After stimulation with IFN-γ, 4T1 cells were further treated with a CatB inhibitor (CA-074-Me)^30^, a NEDD8-activating enzyme inhibitor (MLN4924)^31^, or a 26 S proteasome inhibitor (epoxomicin)^32^. MLN4924 was reported to inhibit the neddylation of E3 ligase (cullin-RING complexes), which needed to be neddylated in order to be active (Fig. 3a)^33^. After such treatments, incubation with SPN_pro_ was no longer able to reduce the green fluorescence signals of FITC-labeled anti-IDO antibodies and the Kyn contents. Thus, the mechanism of SPN_pro_-mediated IDO degradation and Trp-metabolism intervention could be explained as follow: in the presence of CatB, SPN_pro_ was cleaved to induce the release and activation of IPP. The released IPP bound to IDO and brought it to the VHL ligand of E3 ligase. Then, E3 ligase was activated by the NEDD8-activating enzyme that facilitated ubiquitin transfer from E2 ligase to IDO, leading to the polyubiquitination of IDO and subsequent proteasome recruitment. At last, the 26 S proteasome was recruited to IDO and initiated the degradation of IDO, ultimately resulting in inhibited Trp catabolism and Kyn depletion. Meanwhile, IPP was released from the ubiquitin-proteasome system and reactivated to induce persistent IDO degradation in a recycling manner.

Then, the photoactivity and cytotoxicity of SPN_pro_, SPN-1, and SPN-2 were studied in vitro. The intracellular ^1^O_2_ generation was evaluated using 2′,7′-dichlorodihydrofluorescein diacetate (H_2_DCFDA) as the ^1^O_2_ fluorescent turn-on indicator. H_2_DCFDA could be rapidly hydrolyzed by the intracellular esterase and oxidized to fluorescent 2′,7′-dichlorodihydrofluorescein (DCF) in the presence of ^1^O_2_^34^. As shown in Fig. 3g, h, obvious green fluorescence signals of DCF were detected in SPN_pro_-, SPN-1-, or SPN-2-incubated 4T1 cells only after NIR photoirradiation, which were 83.3-, 79.4-, and 71.6-fold stronger than that for the untreated cells, respectively. To further evaluate ^1^O_2_-mediated cytotoxicity of these nanoparticles, 4T1 cells were stained with propidium iodide (PI) to detect the dead cells after NIR photoirradiation. Obvious red fluorescence signals of PI were detected in SPN_pro_-, SPN-1-, or SPN-2-incubated 4T1 cells after NIR photoirradiation, while almost no fluorescence signals of PI were detected in the nanoparticles-treated or photoirradiated cells (Supplementary Fig. 12). Afterwards, the cell viability was studied by a 5-(3-carboxymethoxyphenyl)-2-(4,5-dimethylthiazolyl)-3-(4-sulfophenyl)-tetrazolium (MTS) assay. After incubation with the nanoparticles at different concentrations, the cell viabilities of SPN_pro_-, SPN-1-, and SPN-2-incubated cells were above 90%, suggesting the negligible cytotoxicity of these nanoparticles. After NIR photoirradiation at 808 nm with the power intensity of 0.3 W/cm^2^ for 6 min, the control cells did not exhibit any obvious changes, while SPN_pro_-, SPN-1-, and SPN-2-incubated cells showed increased cytotoxicity in a concentration-dependent manner (Fig. 3i). At the PCB concentration of 40 μg/mL, the cell viabilities of SPN_pro_-, SPN-1-, and SPN-2-incubated cells similarly decreased to ~10%. These data confirmed that SPN_pro_, SPN-1, and SPN-2 possess good phototherapeutic abilities.

### In vivo photo-immunometabolic therapy

**Fig. 4:**
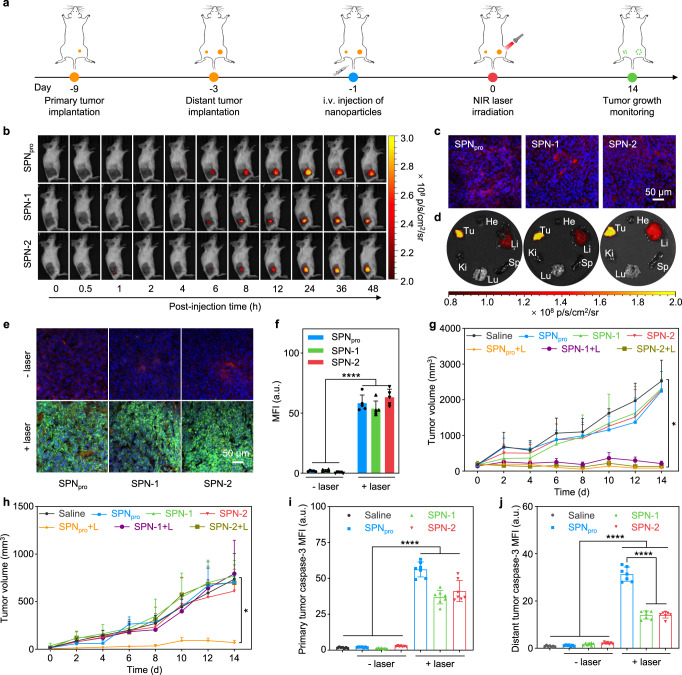
In vivo NIR fluorescence imaging and SPN_pro_-mediated activatable photo-immunometabolic therapy. **a** Schematic illustration of the schedule for tumor model implantation and SPN_pro_-mediated activatable photo-immunometabolic therapy. **b** NIR fluorescence imaging of 4T1-tumor-bearing BALB/c mice at different timepoints after the intravenous injection of SPN_pro_, SPN-1, or SPN-2 (200 μL, [PCB] = 200 μg/mL). **c** Confocal fluorescence images of primary tumors from 4T1-tumor-bearing mice after intravenous injection of SPN_pro_, SPN-1, or SPN-2. Blue fluorescence showed the cell nucleus stained with DAPI, and red fluorescence showed the signals from the PCB core. **d** Ex vivo NIR fluorescence images of tumors and major organs in 4T1-tumor-bearing mice at 48 h after intravenous injection of SPN_pro_, SPN-1, or SPN-2. **e** Confocal fluorescence images of primary tumor tissues from SPN_pro_-, SPN-1-, or SPN-2-injected (200 μL, [PCB] = 200 μg/mL) 4T1-tumor-bearing mice with or without NIR photoirradiation (0.3 W/cm^2^ at 808 nm) for 6 min. Blue fluorescence showed the cell nucleus stained with DAPI, red fluorescence showed the signals from the PCB core, and green fluorescence showed the signals from SOSG. **f** Quantitative analysis of SOSG MFI in primary tumor tissues of mice after different treatments (*n* = 5). *p* < 0.0001. Growth curves of primary tumors (**g**) and distant tumors (**h**) in 4T1-tumor-bearing mice after different treatments (*n* = 5). Saline-treatment versus SPN_pro_-treatment group: *p* = 0.0043 for primary tumors (**g**); *p* = 0.0192 for distant tumors (**h**). Quantification of caspase-3 expression in primary (**i**) and distant (**j**) tumor tissues of 4T1-tumor-bearing mice (*n* = 7). *p* < 0.0001. Statistical significance in **f**, **i**, and **j** was calculated via one-way ANOVA with a Tukey post-hoc test. Statistical significance in **g** and **h** was calculated via two-tailed Student’s *t*-test. *p < 0.032, **p < 0.0021, ***p < 0.0002, and ****p < 0.0001. The mean values and SD are presented. The experiments in **c** and **e** were repeated independently three times with similar results.

SPN_pro_-mediated activatable photo-immunometabolic therapy was further studied in 4T1-tumor-bearing BALB/c mice. 4T1 cells were subcutaneously inoculated in the right flank of mice as the primary tumor; 6 days later, the same amount of 4T1 cells was subcutaneously inoculated in the left flank of mice as the distant tumor (Fig. 4a). After 2 days, 4T1-tumor-bearing mice were intravenously injected with SPN_pro_, SPN-1, or SPN-2 and treated with local NIR photoirradiation. After different treatments, the growths of both primary and distant tumors were monitored for 14 days. To confirm the optimal timepoint for NIR photoirradiation, the accumulation of nanoparticles in the primary tumor tissues was evaluated using NIR fluorescence imaging. After intravenous injection of SPN_pro_, SPN-1, or SPN-2, the fluorescence signals in the primary tumor tissues of these groups gradually increased. At 24 h post-injection time, the fluorescence signals reached a similar maximum MFI, which was about 90-fold higher than that of the background (Fig. 4b, Supplementary Fig. 13). These data indicated that these nanoparticles had similar and effective tumor accumulation abilities owing to their small size (~30 nm) and hydrophilic PEG segment. This was also confirmed by the red fluorescence signals from the PCB core in the primary tumor tissues by immunofluorescence staining (Fig. 4c, Supplementary Fig. 13). In addition, SPN_pro_, SPN-1, and SPN-2 showed similar tissue distribution in major organs (Fig. 4d, Supplementary Fig. 13), showing the highest accumulation in tumor followed by liver.

Photo-immunometabolic therapy was conducted by NIR photoirradiation on the primary tumors at the maximum accumulation timepoint (24 h post-injection of nanoparticles). Notably, the power intensity of the laser was controlled to ensure that the temperature of tumors was below the threshold temperature (43 °C) to minimize photothermal heating effect. The ^1^O_2_ generation of nanoparticles was studied by detecting the fluorescence signals after local injection of SOSG in tumor tissues. There were obvious green fluorescence signals of SOSG and a similar increase (~60-fold) of MFIs in the tumors of SPN_pro_-, SPN-1-, or SPN-2-injected mice after photoirradiation compared to that of the mice treated without photoirradiation. These data confirmed the effective intratumoral ^1^O_2_ generation of these nanoparticles upon NIR photoirradiation (Fig. 4e, f).

The in vivo cancer therapeutic efficiency was studied by monitoring the growths of primary and distant tumors every 2 days after NIR photoirradiation for 6 min. After treatments, the primary tumors in SPN_pro_-, SPN-1-, and SPN-2-injected and photoirradiated mice were completely suppressed, whereas the distant tumors were greatly inhibited only for SPN_pro_-injected and photoirradiated mice, which did not show obvious inhibition for SPN-1- or SPN-2-injected and photoirradiated mice (Fig. 4g, h). The therapeutic effects were further confirmed by immunofluorescence staining and hematoxylin and eosin (H&E) staining. The green fluorescence signals of FITC-labeled anti-caspase-3 antibodies were detected in the primary tumor tissues of SPN_pro_-, SPN-1-, and SPN-2-injected mice after photoirradiation, which were 33.1-, 21.8-, and 24.2-fold higher than the saline-injected mice, respectively (Fig. 4i, Supplementary Fig. 14a). As for the distant tumors, the MFIs of FITC-labeled anti-caspase-3 antibodies in SPN_pro_-injected and photoirradiated mice were increased 34.9-fold relative to that in the saline-injected mice, which were also higher (both 2.2-fold) than that in SPN-1- or SPN-2-injected and photoirradiated mice (Fig. 4j, Supplementary Fig. 14a). In addition, H&E staining showed obvious dead cells in primary and distant tumors of SPN_pro_-injected and photoirradiated mice in contrast to the other groups (Supplementary Fig. 14b), which was consistent with the immunofluorescence staining results. These data suggested that only SPN_pro_-mediated therapy effectively eliminated the primary tumors and inhibit the growth of the distant tumors, indicating its stronger antitumor efficacy relative to SPN-1 and SPN-2.

To evaluate the biosafety of SPNs-mediated photo-immunometabolic therapy, body-weight monitoring and histological analysis of the major organs were conducted. The body weights of saline-, SPN_pro_-, SPN-1-, and SPN-2-injected mice with or without photoirradiation showed no obvious change during 14-day monitoring (Supplementary Fig. 15). Afterwards, H&E staining images of the major organs (heart, liver, lung, and kidney) in SPN_pro_-, SPN-1-, or SPN-2-injected and photoirradiated mice exhibited similar physiological morphologies compared to that in saline-injected mice (Supplementary Fig. 16). These results demonstrated that SPN_pro_, SPN-1, and SPN-2 had high biosafety and biocompatibility.

### In vivo mechanistic studies of photo-immunometabolic therapy

**Fig. 5:**
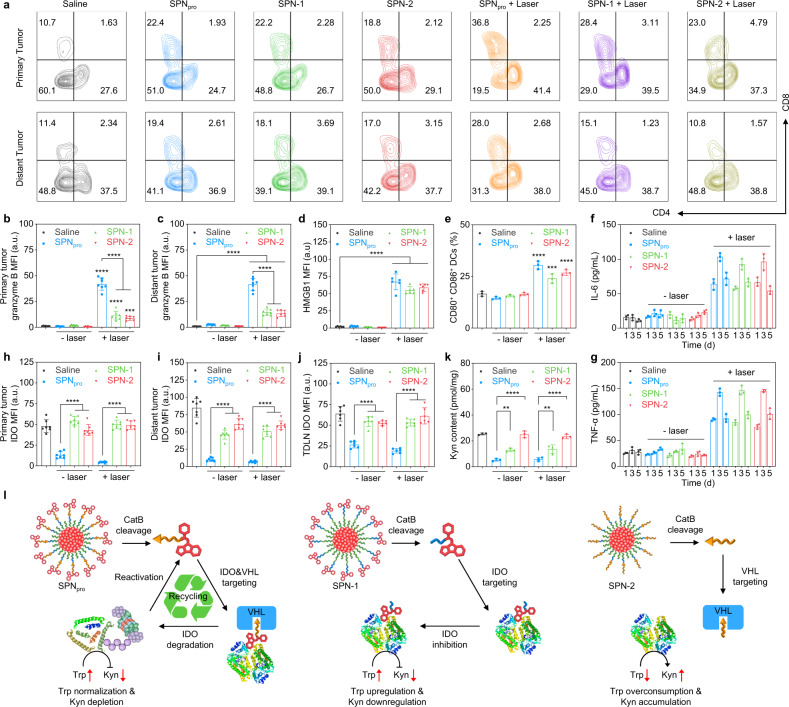
In vivo SPN_pro_-mediated activatable photo-immunometabolic therapy. **a** FACS assay of tumor-infiltrating T lymphocytes (CD8^+^ and CD4^+^) of the primary and distant tumors in 4T1-tumor-bearing mice after different treatments. 4T1-tumor-bearing mice were intravenously injected with saline, SPN_pro_, SPN-1, or SPN-2 (200 μL, [PCB] = 200 μg/mL), and the primary tumors were treated with or without NIR photoirradiation (0.3 W/cm^2^ at 808 nm) for 6 min. Quantification of granzyme B expression in primary (**b**) and distant (**c**) tumor tissues of 4T1-tumor-bearing mice (*n* = 7). *p* < 0.0001; Saline versus SPN-2 + laser: *p* = 0.0004 for primary tumors (**b**). *p* < 0.0001 for distant tumors (**c**). **d** Quantification of HMGB1 expression in the tumor tissues of 4T1-tumor-bearing mice by confocal fluorescence images after different treatments (*n* = 6). *p* < 0.0001. **e** Quantification of matured DCs (CD80^+^ and CD86^+^) from the tumor-draining lymph nodes (TDLNs) in 4T1-tumor-bearing mice after different treatments (*n* = 3). *p* < 0.0001; Saline versus SPN-1 + laser: *p* = 0.0002. In vivo cytokine detection of TNF-α (**f**) and IL-6 (**g**) in sera from mice after different treatments at different timepoints (1, 3, and 5 days) (*n* = 3). **h**–**j** Quantification of IDO expression in primary tumors (**h**), distant tumors (**i**), and TDLNs (**j**) of 4T1-tumor-bearing mice by immunofluorescence staining after different treatments (*n* = 7). *p* < 0.0001. **k** The Kyn content in primary tumors of 4T1-tumor-bearing mice after different treatments (*n* = 3). *p* < 0.0001; SPN_pro_ versus SPN-1: *p* = 0.0038; SPN_pro_ + laser versus SPN-1 + laser: *p* = 0.0041. **l** Schematic illustration of the mechanisms of SPN_pro_-, SPN-1-, and SPN-2-mediated IDO degradation and Trp-metabolism intervention. Statistical significance in **b**, **c**, **d**, **e**, **h**, **i**, **j**, and **k** was calculated via one-way ANOVA with a Tukey post-hoc test. *p < 0.032, **p < 0.0021, ***p < 0.0002, and ****p < 0.0001. The mean values and SD are presented.

To study the mechanism of SPN_pro_-mediated photo-immunometabolic therapy, tumor-infiltrating lymphocytes (TILs) were first detected by collecting the primary and distant tumor tissues and spleens by fluorescence-activated cell sorting (FACS) analysis. The populations of TILs (CD4^+^ and CD8^+^) and cytotoxic TILs (CD8^+^granzyme B^+^) in primary and distant tumor tissues and spleen of SPN_pro_-injected mice were higher than that of SPN-1- or SPN-2-injected mice after NIR photoirradiation (Fig. 5a, Supplementary Fig. 17). Meanwhile, the population of regulatory T cells (CD4^+^Foxp3^+^) and myeloid-derived suppressor cells (CD11b^+^Gr-1^+^) of SPN_pro_-injected mice were lower than that of SPN-1- or SPN-2-injected mice after NIR photoirradiation (Supplementary Fig. 18). In addition, the expression of granzyme B in the tumor tissues was detected to examine the tumor killing effect by cytotoxic TILs. Both the primary and distant tumor tissues of SPN_pro_-injected mice exhibited higher MFIs (3.0- and 4.2-fold, respectively) of FITC-labelled anti-granzyme B antibodies than that for SPN-1- or SPN-2-injected mice (Fig. 5b, c, Supplementary Fig. 19). Moreover, we evaluated the antitumor effects of SPN_pro_ in 4T1-tumor-bearing immunodeficient NOD-Scid *IL2rg*^−/−^ (NSG) mice, which lack functional lymphocytes (including T cells and NK cells) and are defective in immune functions. Both the primary and distant tumors exhibited negligible inhibition effects in SPN_pro_-injected and photoirradiated NSG mice (Supplementary Fig. 20). These data confirmed that SPN_pro_-mediated therapy was dependent on the presence of the immune system and exhibited stronger antitumor T-cell immune responses than SPN-1- or SPN-2-mediated therapy.

To gain insight into the high antitumor T-cell immunity of SPN_pro_-mediated therapy, the corresponding immunological processes including ICD, DC maturation, and cytokine release of nanoparticle-injected 4T1-tumor-bearing mice were investigated and compared. High-mobility group protein B1 (HMGB1) was chosen to be evaluated as the important marker for ICD^35^. The MFIs of FITC-labeled anti-HMGB1 antibodies in the primary tumor tissues of SPN_pro_-, SPN-1-, or SPN-2-injected mice were greatly increased by 30.6-, 25.0-, and 26.7-fold relative to that for the saline-injected mice, respectively (Fig. 5d, Supplementary Fig. 21a). DC maturation was studied by collecting TDLNs to detect the population of CD11c^+^CD80^+^CD86^+^ DCs by flow cytometry analysis. The amounts of matured DCs (CD11c^+^CD80^+^CD86^+^) relative to all DCs (CD11c^+^) in TDLNs for SPN_pro_-, SPN-1-, and SPN-2-injected mice were 30.5, 24.1, and 26.8%, which were 1.8-, 1.5-, and 1.6-fold higher than that of saline-injected mice, respectively (Fig. 5e, Supplementary Figs. 21b, 22). The serum samples of SPN_pro_-, SPN-1-, and SPN-2-injected mice were collected at different timepoints (1, 3, and 5 days) after photoirradiation to detect the cytokines (TNF-α and IL-6) via enzyme-linked immunosorbent assay (ELISA). Both the concentration of TNF-α and IL-6 in SPN_pro_-, SPN-1-, and SPN-2-injected mice reached maximum at 3 days after photoirradiation (Fig. 5f, g). Moreover, the concentration of TNF-α and IL-6 in SPN_pro_-injected mice were 4.7- and 6.4-fold higher than that in saline-injected mice, respectively. These ICD, DC maturation, and cytokine release profiles confirmed that SPN_pro_-, SPN-1-, and SPN-2-mediated therapy enhanced tumor immunogenicity to a similar level, which should be ascribed to their similar phototherapeutic efficiency.

To further verify the enhanced antitumor T-cell immune response of SPN_pro_ relative to other SPNs, the IDO expression and Kyn content in the primary and distant tumor tissues and TDLNs were investigated. Obvious green fluorescence signals of FITC-labelled anti-IDO antibodies were detected in the primary and distant tumor tissues and TDLNs of treated mice (Fig. 5h–j, Supplementary Fig. 23). The MFIs of FITC-labelled anti-IDO antibodies in the primary and distant tumor tissues and TDLNs of SPN_pro_-injected and photoirradiated mice were greatly decreased by 10.9, 12.3, and 3.4 times relative to that of the saline-injected mice, respectively. In addition, the Kyn content in the primary tumor tissues of SPN_pro_-injected mice had obvious decreases before (79.2%) or after (77.2%) photoirradiation relative to that for saline-injected mice (Fig. 5k). Although SPN-1 possessed the ability for IDO inhibition owing to the presence of NLG919 unit, SPN_pro_-injected mice still showed higher decreases of the Kyn contents before (59.6%) or after (57.1%) photoirradiation relative to that for SPN-1-injected mice. These data further confirmed the different mechanisms of SPN_pro_ and SPN-1 for IDO inhibition and subsequent Kyn depletion (Fig. 5l). SPN-1 can inhibit the activity of IDO via occupancy-driven pharmacology, which requires persistent drug binding to the active site of IDO in order to inhibit protein activity. In contrast, SPN_pro_ can directly induce the degradation of IDO in a sustainable manner due to the reactivation and reuse of the active IPP. Thereby, SPN_pro_ mediated more effective IDO degradation and Kyn depletion than SPN-1 and SPN-2 did, leading to better immunometabolic reprogramming and higher antitumor immune response.

## Discussion

Existing PROTAC technology mainly utilizes heterobifunctional binding ligands to induce targeted proteolysis of undruggable oncoproteins for cancer therapy^36–38^, which has not been designed with a biomarker-activatable action. In contrast, SPN_pro_ only activates its PROTAC function in response to a specific cancer biomarker (CatB), realizing targeted proteolysis of an immunometabolism-associated enzyme (IDO). The molecular mechanism of SPN_pro_-mediated IDO degradation was further confirmed using various signaling pathway inhibitors (Fig. 3d–f), showing its high specificity. With its high tumor accumulation and ideal biosafety (Fig. 4b–d, Supplementary Figs. 13, 15, 16), SPN_pro_ enabled targeted degradation of IDO in the tumor of living mice, leading to localized immunometabolism intervention. Thus, SPN_pro_ represents the first smart PROTAC system with high disease specificity to minimize off-target side effects for cancer therapy.

Mechanistically different from inhibitor and genetic approaches, SPN_pro_ reprograms immunometabolic pathways through direct degradation of IDO in a persistent manner (Figs. 3a, 5l), as the activated PROTAC (IPP) can be recycled for repeated proteolysis. It has been validated that SPN_pro_ had the higher IDO degradation (ca. 90%) and Kyn depletion (ca. 70%) efficiencies in IFN-γ-stimulated 4T1 cells (Fig. 3d–f) relative to those for free NLG919 (ca. 10% and ca. 50%) and SPN-1 (ca. 10% and ca. 40%) without PROTAC segment at the same concentration of NLG919 (1 μM). Notably, this Kyn depletion efficiency was also higher than the reported studies on various NLG919-encapsulated nanosystems (ca. 50%) at the same concentration of NLG919 (1 μM) in vitro^39,40^. Moreover, the higher IDO degradation of SPN_pro_ relative to other controls was observed in the tumor of living mice (Fig. 5h–k, Supplementary Fig. 23). Thus, although all three SPNs enhanced tumor immunogenicity to the similar level after NIR phototherapy, SPN_pro_ had the strongest antitumor T-cell immune response (Fig. 5a–c), leading to the most effective inhibition of tumor growth and complete prevention of metastasis (Fig. 4g–j).

In summary, we report a nano-PROTAC (SPN_pro_) that synergizes phototherapeutic function with biomarker-activated protein degradation for photo-immunometabolic cancer therapy. SPN_pro_ represents a unique type of immunotherapeutic nanoagents that specifically block the immunosuppressive metabolism via targeted proteolysis of catabolizing enzyme by the ubiquitin-proteasome system. Such PROTAC design can be generalized for biomarker-activated proteolysis of many other immunometabolism-associated proteins (such as glutaminase, arginase, fatty acid synthase, lactate dehydrogenase, and acetyl-CoA acetyltransferase) by conjugating the corresponding targeting moiety onto the polymer^10^. Thus, our study not only provides a new combinational therapeutic modality, but also opens new opportunities to advance PROTAC in cancer therapy.

## Methods

### Chemicals

All the chemicals were purchased from Sigma–Aldrich unless otherwise stated. 2-Chlorotrityl chloride polystyrene resin, o-benzotriazole-N,N,N’,N’-tetramethyluroniumhexafluoro-phosphate (HBTU), 1-hydroxybenzotriazole (HOBt), *N*-fluorenyl-9-methoxycarbonyl (Fmoc)-protected _L_-amino acids (Fmoc-Gly-OH, Fmoc-Ser(tBu)-OH, Fmoc-Ala-OH, Fmoc-Pro-OH, Fmoc-Tyr(tBu)-OH, Fmoc-Ile-OH, Fmoc-Phe-OH, and Fmoc-Leu-OH) were purchased from GL Biochem. Ltd. (Shanghai, China) and used as received without any purification. SOSG was purchased from Molecular Probes Inc. (Carlsbad, CA, USA). 3-(4,5-Dimethylthiazol-2-yl)-5-(3-carboxymethoxyphenyl)-2-(4-sulfophenyl)-2H-tetrazolium, inner salt (MTS) solution was purchased from Promega Corp. (Madison, WI, USA). Dulbecco’s modified eagle medium (DMEM) with L-glutamine was purchased from Lonza. Trypsin–EDTA (0.05%), penicillin–streptomycin (10,000 U/mL), fetal bovine serum (FBS), ACK lysis, type I collagenase, and type IV collagenase were purchased from Gibco. MLN4924, IDO antibody (Catalog no. ab106134, dilution: 1:50), granzyme B antibody (ab255598, dilution: 1:200), and secondary antibody Alexa Fluor 488 conjugated goat anti-rabbit IgG H&L (ab150077, dilution: 1:500) were purchased from Abcam Inc. (Cambridge, CA, USA). HMGB1 antibody (Catalog no. 3935 S, dilution: 1:100) and cleaved caspase-3 antibody (Catalog no. 9661 L, dilution: 1:500) were purchased from Cell Signaling Technology. ELISA kits for TNF-α and IL-6 detection, PE-CD11b antibody (Catalog no. 101208, dilution: 1:80), BV605-Gr-1 antibody (Catalog no. 108440, dilution: 1:40), AF647-Foxp3 antibody (Catalog no. 126408, dilution: 1:50), APC anti-mouse CD11c (Catalog no. 117310, dilution: 1:80), FITC anti-mouse CD80 (Catalog no. 104706, dilution: 1:50), PE anti-mouse CD86 (Catalog no. 105008, dilution: 1:20), FITC anti-mouse CD3 (Catalog no. 100204, dilution: 1:50), APC anti-mouse CD8a (Catalog no. 100712, dilution: 1:80), PE anti-mouse CD4 (Catalog no. 130310, dilution: 1:80), and purified anti-mouse CD16/32 (Catalog no. 156604, dilution: 1:200) were purchased from Biolegend. HO-PEG_2000_-COOH was purchased from JenKem Technology USA Inc. NLG919 was purchased from D&C Chemicals.

### Material characterization

Proton nuclear magnetic resonance (^1^H NMR) spectra were recorded on a Bruker Avance II 300 MHz system (Bruker Physik AG, Germany). Electrospray ionization-mass spectrometry (ESI-MS) spectra were conducted with a Thermo Finnigan Polaris Q quadrupole ion trap mass spectrometer (Thermo Fisher Corporation) equipped with a standard ESI source. Absorption and fluorescence spectra were measured on a UV-2450 spectrophotometer (Shimadzu, Japan) and a Fluorolog 3-TCSPC spectrofluorometer (Horiba Jobin Yvon), respectively. DLS measurements were performed on a Malvern Nano-ZS Particle Sizer (Malvern Instruments, Southborough, UK). TEM images were captured using a JEM 1400 transmission electron microscope (JEOL, Tokyo, Japan). HPLC analyses and purification were performed on an Agilent 1260 system using methanol (MeOH)/water (H_2_O) as the eluent. Confocal images were captured using a LSM800 confocal laser scanning microscope (Carl Zeiss, Germany). Flow cytometry assay was performed on Fortessa X20 (BD Biosciences). In vivo animal fluorescence images were captured using an IVIS imaging system (IVIS-CT machine, PerkinElmer). Tissues were cut into sections using a cryostat (Leica). The tissue sections were examined on a Nikon ECLIPSE 80i microscope (Nikon Instruments). NMR spectra were analyzed using Mestre Nova LITE v5.2.5-4119 software (Mestre lab Research S.L.).

### Synthesis of carbonylated NLG919

A mixture of NLG919 (564 mg, 2 mmol), succinic anhydride (240 mg, 2.4 mmol), and 4-dimethylaminopyridine (24.4 mg, 0.2 mmol) in anhydrous dichloromethane (DCM, 20 mL) was stirred at room temperature for 48 h. After the reaction mixture was concentrated under reduced pressure, it was washed with ammonium chloride solution and extracted by DCM. The organic layer was dried with anhydrous sodium sulphate and concentrated under vacuum to afford carbonylated NLG919 as a white crystalline solid. TLC (silica gel, DCM), R_f_ = 0.5. ^1^H NMR (300 MHz, CDCl_3_): δ 0.85–1.79 (m, 10H), 2.36 (m, 1H), 2.72 (m, 4H), 3.46 (s, 1H), 5.32 (m, 2H), 6.46 (m, 1H), 7.08–8.15 (m, 4H). ESI-MS (*m/z*): calc: 382.2, found [M + H]^+^: 383.2.

### Synthesis of IPCP

2-Chlorotrityl chloride resin (1.14 mmol/g) was soaked in anhydrous dimethylformamide (DMF) for 1 h. Then a mixture of Fmoc-Gly-OMe (4 equiv.) and diisopropylethylamine (DIEA, 10 equiv.) was dissolved in DMF and added into the resins for stirring 3 h under a N_2_ atmosphere. After being washed thrice with DMF to remove the unreacted mixture solution, the resins were reacted with a mixture of MeOH and DIEA in DMF (v/v/v = 1:2:7) for 30 min to cap the unreacted groups. Then, the resins were incubated with 20% piperidine in DMF (v/v) for 15 min twice to remove the Fmoc protecting groups for further amide condensation reaction. The next amino acid couplings were conducted by reacting a mixture of Fmoc-protected amino acids (3 equiv.), HBTU (3.6 equiv.), HOBT (3.6 equiv.), and DIEA (7.5 equiv.) with the resins for 2 h. Kaiser reagent was further used to confirm the coupling efficiency. After repeated amide condensation reaction with Fmoc-protected amino acids, the resins were reacted with a mixture of carbonylated NLG919 (2 equiv.), HBTU (2.4 equiv.), HOBT (2.4 equiv.), and DIEA (5 equiv.) overnight. After being washed with DMF (4 times), MeOH (4 times), and DCM (4 times), the resins were dried under vacuum for 30 min. Finally, the chimeric peptide IPCP was cleaved from the resins with a mixture of trifluoroacetic acid (TFA)/H_2_O/1,2-ethanedithiol (v/v/v = 95:2.5:2.5) for 1.5 h at room temperature. After being collected and concentrated under reduced pressure, the filtrate was dropped into cold anhydrous ether to obtain IPCP as precipitates. ^1^H NMR (300 MHz, CD_3_OD): δ 0.88 (m, 24H), 1.22–1.72 (m, 23H), 1.81–2.30 (m, 12H), 2.83–3.18 (m, 6H), 3.43–4.04 (m, 21H), 4.21–4.67 (m, 13H), 6.61–7.65 (m, 15H). ESI-MS (*m/z*): calc: 1826.9, found [M + 2H]^2+^: 914.8.

For comparison, the control peptides ICP and PCP were also prepared in a similar method. ICP was synthesized without the PROTAC segment. ^1^H NMR (300 MHz, CD_3_OD): δ 0.84–1.37 (m, 19H), 2.30–3.01 (m, 8H), 3.40–3.98 (m, 10H), 7.04–7.92 (m, 11H). ESI-MS (*m/z*): calc: 813.4, found [M + H]^+^: 814.4. PCP was synthesized without the NLG919 unit. ^1^H NMR (300 MHz, CD_3_OD): δ 0.70–1.34 (m, 24H), 1.37–2.17 (m, 20H), 2.82–3.24 (m, 5H), 3.41–3.69 (m, 3H), 4.08–3.69 (m, 13H), 4.12–4.72 (m, 11H), 6.55–7.38 (m, 9H). ESI-MS (*m/z*): calc: 1504.7, found [M + 2H]^2+^: 753.7.

### Synthesis of PEG-IPCP

Amino-PEG-alkyne (Mw = 2000) was synthesized according to a previous study^24^. HO-PEG-COOH (Mw = 2000, 2 g, 1 mmol) and NaH (100 mg, 4.2 mmol) were dissolved in dry tetrahydrofuran (THF) and stirred at 0 °C for 1 h. Then, propargyl bromide (120 μL, 1.52 mmol) and NaH (50 mg, 2.1 mmol) were added and stirred overnight. The reaction solution was filtered to remove precipitates, and the filtrate was concentrated via rotary evaporation. Afterwards, the crude product was precipitated in cold diethyl ether and collected via centrifugation, followed by dialysis and lyophilization to obtain carboxyl-PEG-alkyne. Carboxyl-PEG-alkyne (500 mg, 0.25 mmol), EDC (120 mg, 0.625 mmol), and NHS (72 mg, 0.625 mmol) were dissolved in anhydrous THF, and then stirred at room temperature for 1 h. Afterwards, ethylenediamine (160 μL, 2.5 mmol) was added and then stirred for 2 days. After reaction, THF was removed by rotary evaporation, and the residues were purified by dialysis and lyophilization to obtain amino-PEG-alkyne. ^1^H NMR (300 MHz, CDCl_3_): δ 4.19 (s, 2H), 4.04 (s, 2H), 3.87 (m, 2H), 3.63 (m, 176H), 2.44 (m, 3H).

A mixture of IPCP (50 mg, 0.03 mmol), EDC (14 mg, 0.075 mmol), and NHS (8 mg, 0.075) was dissolved in 10 mL THF and stirred at room temperature for 1 h. Then, the solution was added into 5 mL THF solution containing amino-PEG-alkyne (100 mg, 0.05 mmol) and stirred at room temperature for another 48 h. The obtained PEG-IPCP was further dialyzed against H_2_O for 3 days using a dialysis membrane with MWCO of 2000 and then lyophilized in vacuum. ^1^H NMR (300 MHz, CDCl_3_): δ 0.60–1.41 (m, 19H), 1.46–2.74 (m, 23H), 3.28–3.52 (m, 3H), 3.65 (m, 180H), 3.79–4.42 (m, 10H), 6.49–7.22 (m, 2H), 7.32–8.81 (m, 4H).

For comparison, the control polymers PEG-ICP and PEG-PCP were also prepared in a similar method. PEG-ICP was synthesized by reacting amino-PEG-alkyne with ICP. ^1^H NMR (300 MHz, CDCl_3_): δ 0.74–0.98 (m, 6H), 0.99–1.79 (m, 17H), 1.84–3.19 (m, 17H), 3.26–3.48 (m, 3H), 3.65 (m, 180H), 3.83–4.35 (m, 5H). PEG-PCP was synthesized by reacting amino-PEG-alkyne with PCP. ^1^H NMR (300 MHz, CDCl_3_): δ 0.66–1.06 (m, 3H), 1.08–1.40 (m, 3H), 1.39–2.27 (m, 4H), 2.28–2.51 (m, 2H), 2.53–3.50 (m, 10H), 3.65 (m, 180H), 3.81–4.62 (m, 11H), 6.62–7.05 (m, 1H), 7.42–8.45 (m, 3H).

### Synthesis of PCB-PEG-IPCP

PCB-N_3_ and mPEG-alkyne (Mw = 1000) were synthesized according to the previous study^28^. mPEG-OH (Mw = 1000, 1 g, 1 mmol) and NaH (100 mg, 4.2 mmol) were dissolved in dry tetrahydrofuran (THF) and stirred at 0 °C for 1 h. Then, propargyl bromide (120 μL, 1.52 mmol) and NaH (50 mg, 2.1 mmol) were added and stirred overnight. The reaction solution was filtered to remove precipitates and the filtrate was concentrated via rotary evaporation. Afterwards, the crude product was precipitated in cold diethyl ether and collected via centrifugation, followed by dialysis and lyophilization to obtain mPEG-alkyne. ^1^H NMR (300 MHz, CDCl_3_): δ 4.21 (s, 2H), 3.65 (m, 88H), 3.38 (s, 3H), 2.45 (s, 1H).

2,6-Dibromo-4,4-bis(6-bromohexyl)-4H-cyclopenta[2,1-b:3,4-b’]dithiophene (50 mg, 0.075 mmol), 4,7-bis(4,4,5,5-tetramethyl-1,3,2-dioxaborolan-2-yl)benzo[c] [1,2,5]thiadiazole (29 mg, 0.075 mg), Pd(PPh_3_)_4_ (5.5 mg, 0.052 mmol), and K_2_CO_3_ (103.7 mg, 0.75 mmol) were added to a 50-mL Schlenk tube. Then, 2.5 mL toluene with methyltrioctylammonium chloride (1 mg) and 1.25 mL water were injected into the Schlenk tube. The mixture was degassed 3 times with freeze-pump-thaw circles and then stirred at 100 °C under nitrogen atmosphere for 1 h. Thereafter, the solvent was removed via rotary evaporation, and the precipitates were redissolved in dichloromethane, followed by three times of washing with water. Subsequently, the obtained PCB-Br was precipitated in methanol, washed 3 times with methanol and dried under vacuum. PCB-Br (12 mg) dissolved in 4.8 mL THF was added into 2.4 mL DMF containing sodium azide (4.8 mg). Then, the mixture was stirred at room temperature for 12 h. After that, the solvent was removed under reduced pressure and the obtained PCB-N_3_ was redissolved in dichloromethane, washed with water for three times. The organic phase was concentrated and precipitated in methanol, followed by three times of washing with methanol and dried under vacuum.

A mixture of N,N,N’,N”,N”’-pentamethyldiethylenetriamine (PMDETA, 13.4 mg), PCB-N_3_ (2 mg), CuBr (2.2 mg), mPEG-alkyne (12 mg), and PEG-IPCP (12 mg) in 3 mL THF was stirred at room temperature under N_2_ atmosphere for 48 h. Then the solvent was evaporated under reduced pressure, and the obtained PCB-PEG-IPCP was redissolved in water and dialyzed against water using a dialysis membrane with MWCO of 10,000 for 3 days.

For comparison, the control polymers PCB-PEG-ICP and PCB-PEG-PCP were also prepared in a similar method. PCB-PEG-ICP was synthesized by reacting PCB-N_3_ with PEG-ICP. PCB-PEG-PCP was synthesized by reacting PCB-N_3_ with PEG-PCP.

### In vitro photodynamic studies

PBS solutions (1 mL) containing SPN_pro_, SPN-1, or SPN-2 ([PCB] = 20 μg/mL) were mixed with 1 μL SOSG probe (500 μM). Then the solutions were irradiated with NIR laser (0.3 W/cm^2^ at 808 nm) for 7 min. The fluorescence intensities of various samples at 520 nm were recorded every 1 min during NIR photoirradiation using a Fluorolog 3-TCSPC spectrofluorometer termed as F. The fluorescence intensities of the samples at 520 nm without NIR photoirradiation were also recorded as F_0_. At last, the enhancement of fluorescence intensities of each sample was calculated as F/F_0_, indicating the generation of ^1^O_2_.

### In vitro CatB-specific activation studies

The buffer solutions (200 μL) containing SPN_pro_, SPN-1, or SPN-2 ([PCB] = 40 μg/mL) were incubated with CatB (0.2 U/mL) for 6 h. Then the solutions with or without CatB incubation were purified by filtration through a 0.22 μm polyvinylidene fluoride syringe driven filter (Millipore), followed by HPLC analysis.

### In vitro cellular uptake assay

4T1 cancer cells were purchased from American Type Culture Collection (ATCC) and cultured in DMEM cell culture medium supplemented with 10% FBS and 1% antibiotics (penicillin−streptomycin, 10,000 U/mL) at 37 °C and 5% CO_2_. The cells were seeded into confocal cell culture dishes at a density of 5 × 10^4^ cells/dish and cultured overnight for cell attachment. Then the cells were incubated with SPN_pro_, SPN-1, or SPN-2 ([PCB] = 20 μg/mL) for 24 h. After washed three times with PBS to remove free nanoparticles, the cells were stained with DAPI, and the fluorescence images of cells were captured using a LSM800 confocal laser scanning microscope (Carl Zeiss, Germany).

### In vitro lysosome colocalization assay

4T1 cancer cells were cultured in DMEM cell culture medium supplemented with 10% FBS and 1% antibiotics (penicillin−streptomycin, 10,000 U/mL) at 37 °C and 5% CO_2_. The cells were seeded into confocal cell culture dishes at a density of 5 × 10^4^ cells/dish and cultured overnight for cell attachment. Then the cells were incubated with SPN_pro_, SPN-1, or SPN-2 ([PCB] = 20 μg/mL) for 24 h. After washed three times with PBS to remove free nanoparticles, the cells were stained with DAPI and the lysosome tracker (Green DND-26), and the fluorescence images of cells were captured using a LSM800 confocal laser scanning microscope (Carl Zeiss, Germany).

### In vitro IDO expression assay

4T1 cancer cells were seeded into confocal cell culture dishes at a density of 5 × 10^4^ cells/dish and cultured overnight for cell attachment. Then the cells were stimulated with IFN-γ for 12 h and incubated with NLG919, IPP, SPN_pro_, SPN-1, or SPN-2 ([PCB] = 20 μg/mL, [NLG919] = 1 μM) for 12 h. After washed three times with PBS to remove free samples, the cells were stained with DAPI and anti-IDO antibodies, and the fluorescence images of cells were captured using a LSM800 confocal laser scanning microscope (Carl Zeiss, Germany).

For the mechanistic study of PROTAC-mediated IDO degradation, IFN-γ-stimulated 4T1 cancer cells were further treated with CA-074-Me, MLN4924, or epoxomicin for 6 h. Then the cells were incubated with or without SPN_pro_ ([PCB] = 20 μg/mL) for 12 h. After washed three times with PBS to remove free samples, the cells were stained with DAPI and anti-IDO antibodies, and the fluorescence images of cells were captured using a LSM800 confocal laser scanning microscope (Carl Zeiss, Germany).

### In vitro Kyn content measurement

4T1 cancer cells were seeded in 6-well cell culture plates at a density of 3 × 10^5^ cells/well and then cultured overnight for cell attachment. Then the cells were stimulated with IFN-γ for 12 h and further treated with or without CA-074-Me, MLN4924, or epoxomicin for 6 h, followed by incubation with NLG919, IPP, SPN_pro_, SPN-1, or SPN-2 ([PCB] = 20 μg/mL, [NLG919] = 1 μM) for 12 h. After that, 1 mL of the supernatant in each well was collected and incubated with 100 μL of 30% trichloroacetic acid at 50 °C for 30 min. Then the supernatant in each well was collected for HPLC analysis to quantify the Kyn contents.

### Intracellular ROS generation measurement

4T1 cancer cells were seeded in confocal cell culture dishes at a density of 5 × 10^4^ cells/dish and then cultured overnight for cell attachment. Then the cells were incubated with SPN_pro_, SPN-1, or SPN-2 ([PCB] = 20 μg/mL) for 12 h. Afterwards, the cells were added with H_2_DCFDA probe and cultured for 30 min. The treated cells were irradiated with NIR laser (0.3 W/cm^2^ at 808 nm) for 6 min. The cells were washed three times with PBS, and the fluorescence images of cells were captured using a LSM800 confocal laser scanning microscope (Carl Zeiss, Germany).

### In vitro cell viability assay

4T1 cancer cells were seeded in 96-well cell culture plates at a density of 1 × 10^4^ cells/well and then cultured overnight for cell attachment. Then the cells were incubated with SPN_pro_, SPN-1, or SPN-2 at different concentrations ([PCB] = 10, 20, 30, and 40 μg/mL) for 12 h. The cells without nanoparticle incubation were used as control. Then the cells were irradiated with NIR laser (0.3 W/cm^2^ at 808 nm) for 6 min. The cells with or without photoirradiation were further cultured for 12 h and then incubated with MTS in DMEM cell culture medium for 4 h. The absorbance at 490 nm (A) of each well was measured using a SpectraMax M5 microplate. The relative cell viability was calculated as follows: cell viability = (A in treated group/A in control group) × 100%.

### Mouse tumor model implantation

Animal experiments were performed in compliance with Guidelines for Care and Use of Laboratory Animals of the Nanyang Technological University-Institutional Animal Care and Use Committee (NTU-IACUC) and approved by the Institutional Animal Care and Use Committee (IACUC) for Animal Experiment, Singapore. Six-week-old female BALB/c mice and immunodeficient NSG mice were purchased from InVivos (Singapore). Mice were housed in a temperature controlled (22 °C) room with 12 h dark-light cycles (0700 h on and 1900 h off) and 40–70% humidity. 4T1 cancer cells suspended in DMEM cell culture medium were subcutaneously inoculated into the right flank (primary tumors) of each mouse (2 × 10^6^ cells/mouse). Six days later, the same amounts of the cells were subcutaneously inoculated in the left flank (distant tumors) of the same mouse. The mice were used for in vivo NIR fluorescence imaging and therapy after 8 days of growth for primary tumors.

### In vivo tumor NIR fluorescence imaging

4T1-tumor-bearing BALB/c mice were randomly divided into three groups (*n* = 3). Mice in each group were intravenously injected with 200 μL PBS solutions containing SPN_pro_, SPN-1, or SPN-2 ([PCB] = 200 μg/mL). At before (0 h) and different post-injection timepoints, the mice were imaged using an IVIS fluorescence imaging system with the excitation wavelength at 710 nm and the emission wavelength at 820 nm. Fluorescence intensity of primary tumor in each mouse was further quantified using Living Image software.

### Ex vivo biodistribution

At 48 h post-injection of various nanoparticles, 4T1-tumor-bearing mice were euthanized, then hearts, livers, spleens, lungs, kidneys, and the primary tumors were extracted and imaged using an IVIS fluorescence imaging system with the excitation wavelength at 710 nm and the emission wavelength at 820 nm. Fluorescence intensity quantification of these tissues was further performed using Living Image software (*n* = 3).

### In vivo tumor ^1^O_2_ generation

4T1-tumor-bearing mice were randomly divided into three groups (*n* = 3). The mice in each group were intravenously injected with 200 μL PBS solutions containing SPN_pro_, SPN-1, or SPN-2 ([PCB] = 200 μg/mL). At 24 h post-injection timepoint, primary tumor of each mouse was locally injected with 20 μL SOSG probe (100 μM) and subsequently irradiated with NIR laser (0.3 W/cm^2^ at 808 nm) for 6 min. After that, the mice were euthanized, and the primary tumors were collected, fixed with 4% paraformaldehyde, and cut into 10-μm sections. Then the tumor sections were stained with DAPI, and the fluorescence images of the stained sections were captured using a LSM800 confocal laser scanning microscope. The fluorescence intensity of SOSG in each image was quantified using software ImageJ.

### In vivo cancer photo-immunometabolic therapy

4T1-tumor-bearing BALB/c mice were randomly divided into seven groups (*n* = 7). The mice in each group were intravenously injected with 200 μL saline or PBS solutions containing SPN_pro_, SPN-1, or SPN-2 ([PCB] = 200 μg/mL). At 24 h post-injection timepoint, primary tumor of each mouse was irradiated with or without NIR laser (0.3 W/cm^2^ at 808 nm) for 6 min. Then the sizes of primary and distant tumors and the body weights of mice were measured every 2 days for 14 days. The tumor volume was calculated as follows: volume = (tumor length) × (tumor width)^2^/2. After 14 days of different treatments, the mice in each group were euthanized, and the tumor tissues and TDLNs were collected for subsequent H&E and immunofluorescence staining (caspase-3, HMGB1, and granzyme B).

In addition, 4T1-tumor-bearing NSG mice were randomly divided into three groups (*n* = 5). The mice in each group were intravenously injected with 200 μL saline or PBS solutions containing SPN_pro_ ([PCB] = 200 μg/mL). At 24 h post-injection timepoint, primary tumor of each mouse was irradiated with or without NIR laser (0.3 W/cm^2^ at 808 nm) for 6 min. Then the sizes of primary and distant tumors and the body weights of mice were measured every 2 days for 14 days. The tumor volume was calculated as follows: volume = (tumor length) × (tumor width)^2^/2.

### Histological studies

After 14 days of different treatments, 4T1-tumor-bearing mice in each group (*n* = 3) were euthanized, and the tumors and major organs (heart, liver, lung, and kidney) were collected and fixed with 4% paraformaldehyde for H&E staining. The stained tissue sections were examined on a Nikon ECLIPSE 80i microscope (Nikon Instruments).

### In vivo evaluation of T cell and myeloid-derived suppressor cell population

After 14 days of different treatments, the mice in each group were euthanized, and the tumor tissues were collected and digested with 1 mg/mL type I collagenase, 100 μg/mL type IV collagenase, and 100 μg/ml DNase I for 2 h at 37 °C, then the tissues were filtered through a 70 μm nylon cell strainer. Then the red blood cells of the obtained tumor single-cell suspension were removed using ACK lysis, and the cells were washed thrice with cell staining buffer. After that, the cells were incubated with anti-CD16 antibody for 10 min. For the cell surface antigen staining, the cells were stained with anti-CD45, anti-CD3, anti-CD4, anti-CD8a, anti-Gr-1, and anti-CD11b antibodies for another 30 min. For the intracellular antigen staining, the cells were fixed and permeabilized, then stained with anti-Foxp3 and anti-granzyme B antibodies for another 30 min. After washed thrice with cell staining buffer, the cells were analyzed by Fortessa X20 (BD Biosciences).

### In vivo evaluation of DC maturation and cytokine release

After 5 days of different treatments, the mice in each group were euthanized, and the TDLNs were collected and directly ground. Then the obtained single cells were incubated with anti-CD16 antibody for 10 min, and then stained with anti-CD11c, anti-CD80, and anti-CD86 antibodies for another 30 min. After washed three times, the cells were analyzed by Fortessa X20 (BD Biosciences).

After 1, 3, or 5 days of different treatments, the mice in each group were euthanized, and the blood samples were collected for the detection of TNF-α and IL-6 by ELISA.

### In vivo evaluation of IDO expression and Kyn content

After 14 days of different treatments, the mice in each group were euthanized, and the tumor tissues and TDLNs were collected for immunofluorescence staining (IDO). Then the tumor tissues were homogenized using a homogenizer. The obtained homogenate was incubated with 30% trichloroacetic acid at 50 °C for 30 min. Then the supernatant was collected for HPLC analysis to quantify the Trp and Kyn contents.

### Statistical analysis

The data in all experiments were expressed as mean ± SD. Statistical calculation of experimental data was performed using the one-way ANOVA with a Tukey post-hoc test or two-tailed Student’s *t*-test. For all tests, *p*-values less than 0.1234 were considered statistically significant; *p < 0.032, **p < 0.0021, ***p < 0.0002, and ****p < 0.0001. All statistical calculations were performed using GraphPad Prism 8.0.

### Reporting summary

Further information on research design is available in the Nature Research Reporting Summary linked to this article.

## Supplementary information

Supplementary Information

Reporting Summary

## Data Availability

All the data supporting the findings of this study are available within the article and its supplementary information files and from the corresponding author upon reasonable request. A reporting summary for this article is available as a Supplementary Information file.
