# Supplementary material for: Semiconducting polymer nano-PROTACs for activatable photo-immunometabolic cancer therapy
Source: Nat Commun. 2021 May 18;12:2934. doi: 10.1038/s41467-021-23194-w (PMC8131624; doi:10.1038/s41467-021-23194-w)
Supplement: Supplementary file 1 — Supplementary Information [file 41467_2021_23194_MOESM1_ESM.pdf]

Supplementary Information for

Semiconducting Polymer Nano-PROTACs for Activatable Photo-immunometabolic Cancer Therapy

*Chi Zhang[1], Ziling Zeng[1], Dong Cui[1], Shasha He[1], Yuyan Jiang[1], Jingchao Li[1], Jiaguo Huang[1], and Kanyi Pu[1,2]**

[1] School of Chemical and Biomedical Engineering, Nanyang Technological University, 70 Nanyang Drive, 637457, Singapore.

[2] Division of Chemistry and Biological Chemistry, School of Physical and Mathematical Sciences, Nanyang Technological University, 21 Nanyang Link, 637371, Singapore.

*E-mail: kypu@ntu.edu.sg

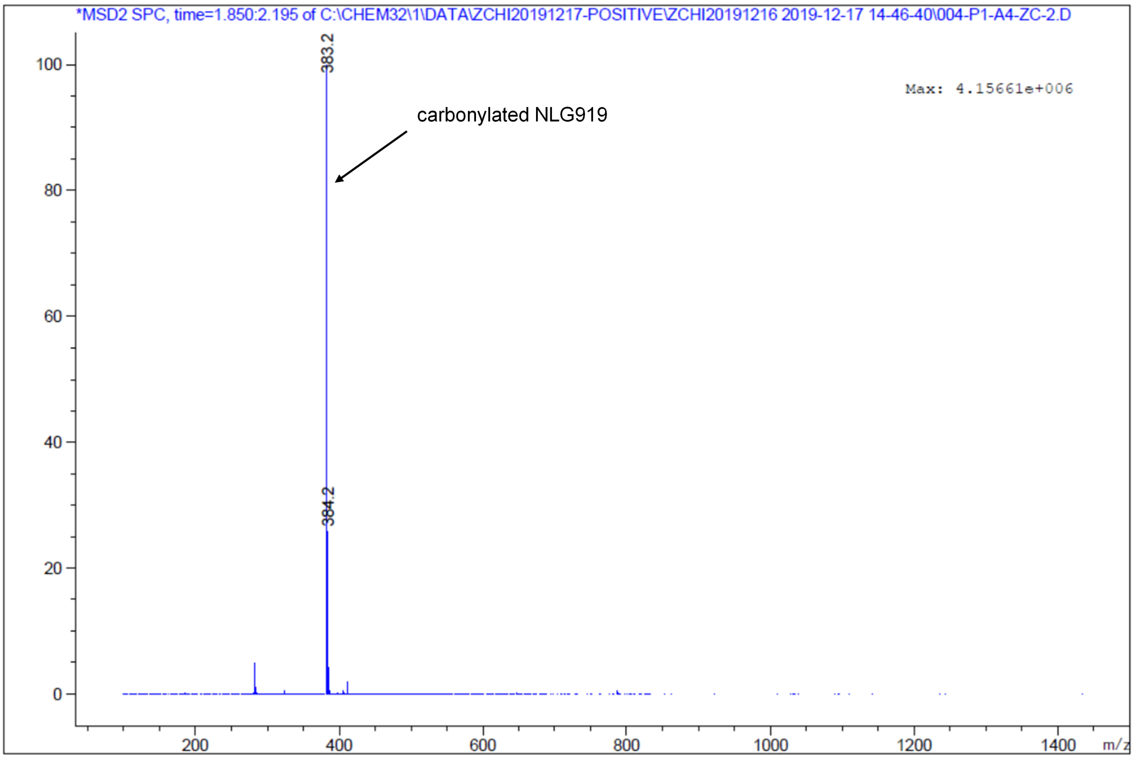

Supplementary Figure 1. ESI-MS of the carbonylated NLG919.

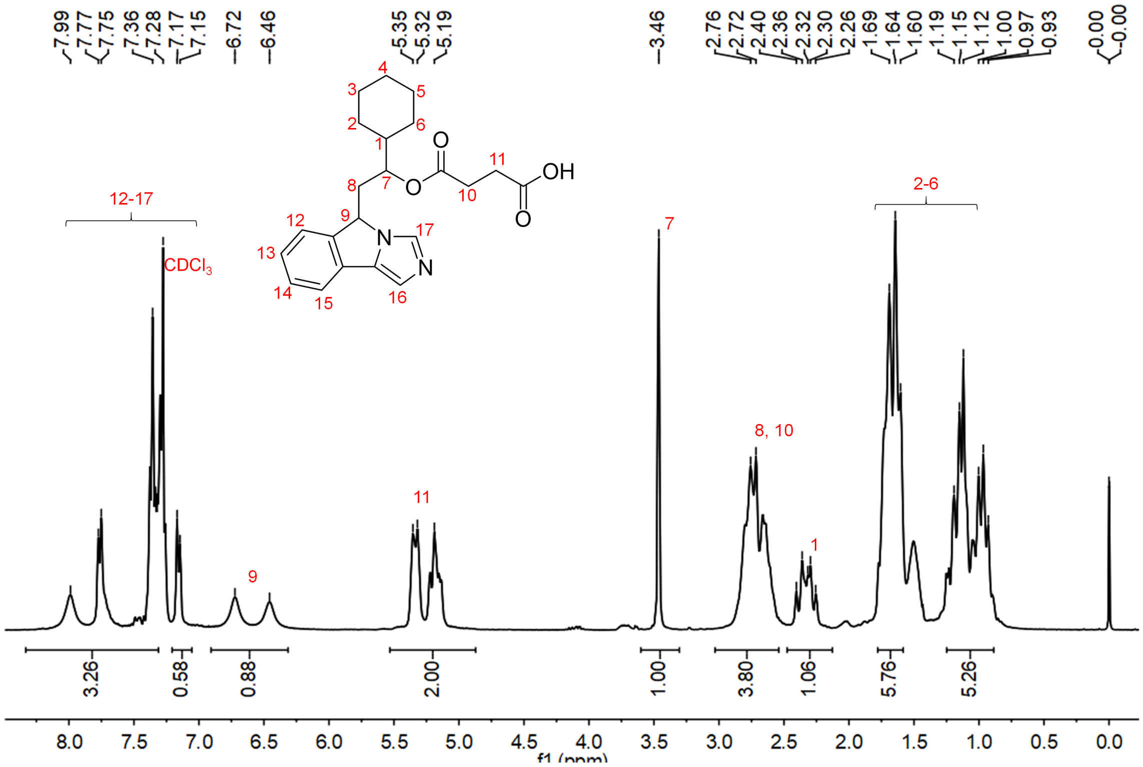

Supplementary Figure 2. ^{1}H NMR spectrum of the carbonylated NLG919 in CD_3Cl.

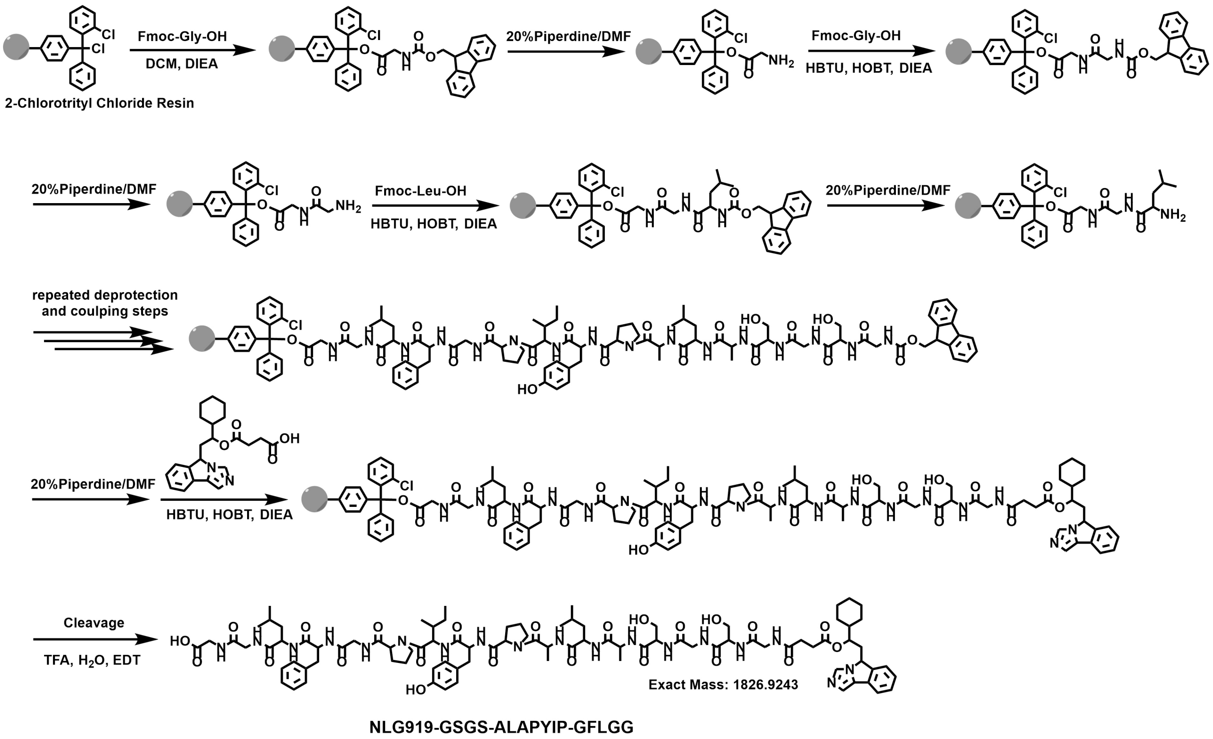

Supplementary Figure 3. Synthesis route of the chimeric peptide IPCP.

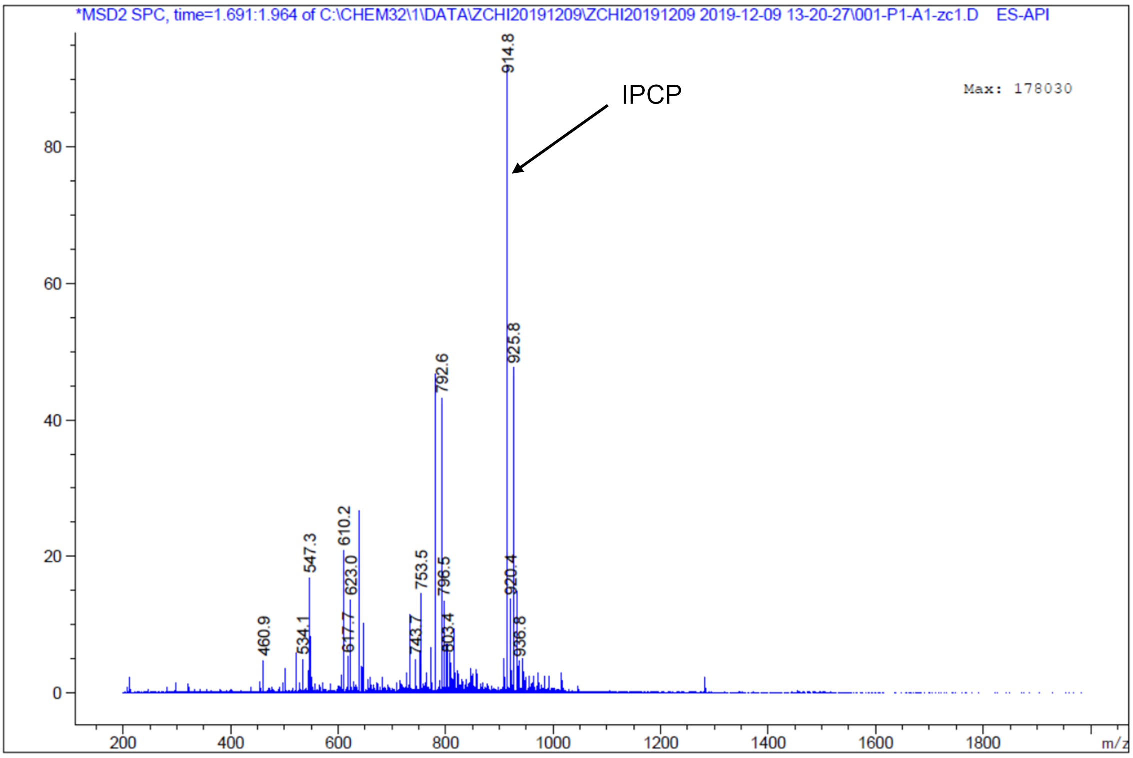

Supplementary Figure 4. ESI-MS of the chimeric peptide IPCP.

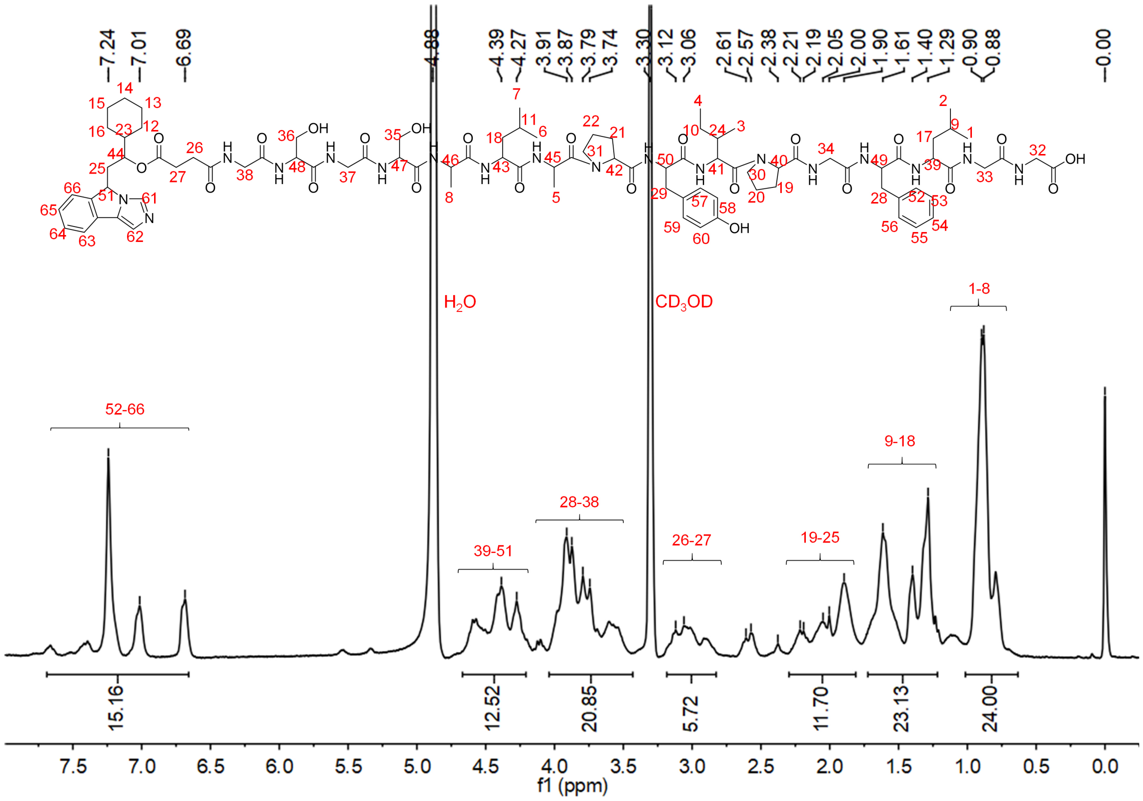

Supplementary Figure 5. ^1H NMR spectrum of the chimeric peptide IPCP in CD_3OD.

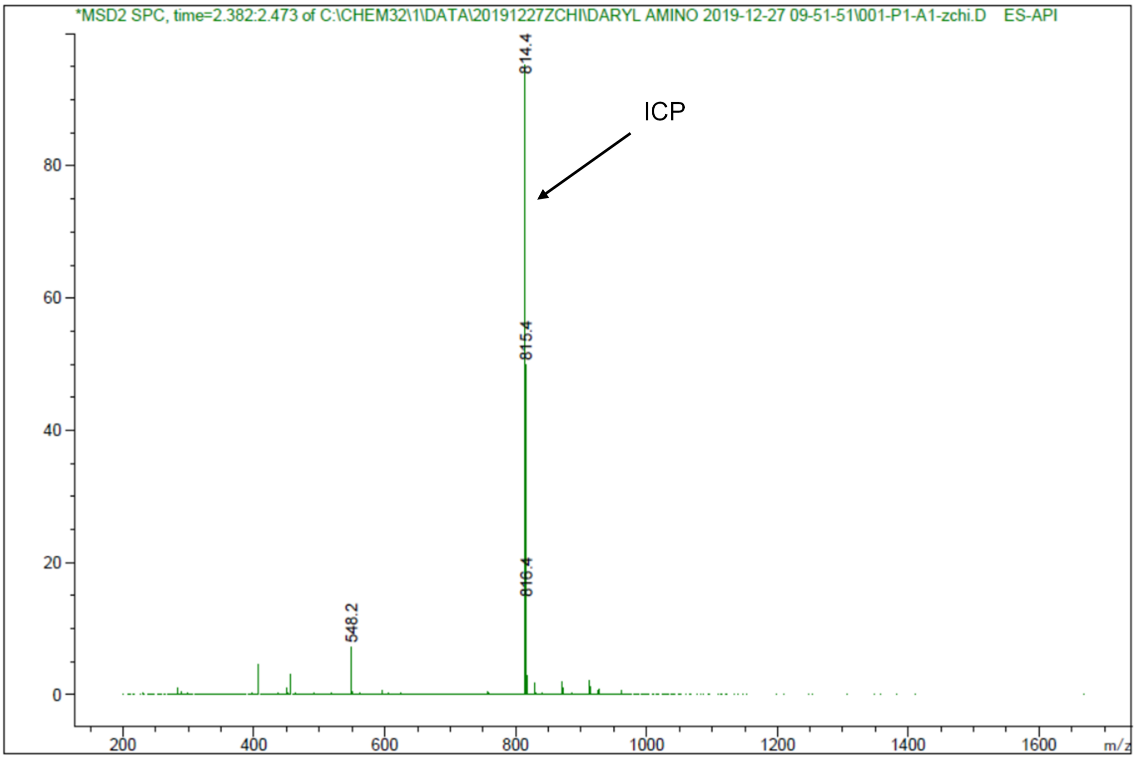

Supplementary Figure 6. ESI-MS of the chimeric peptide ICP.

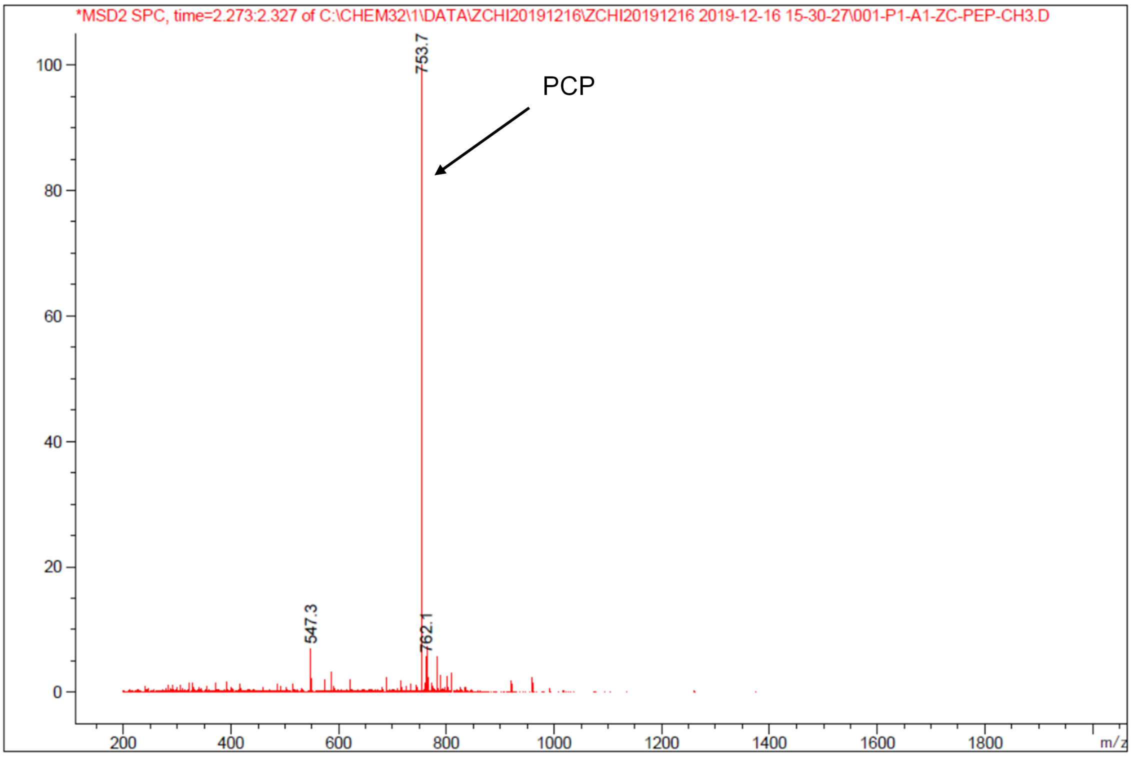

Supplementary Figure 7. ESI-MS of the chimeric peptide PCP.

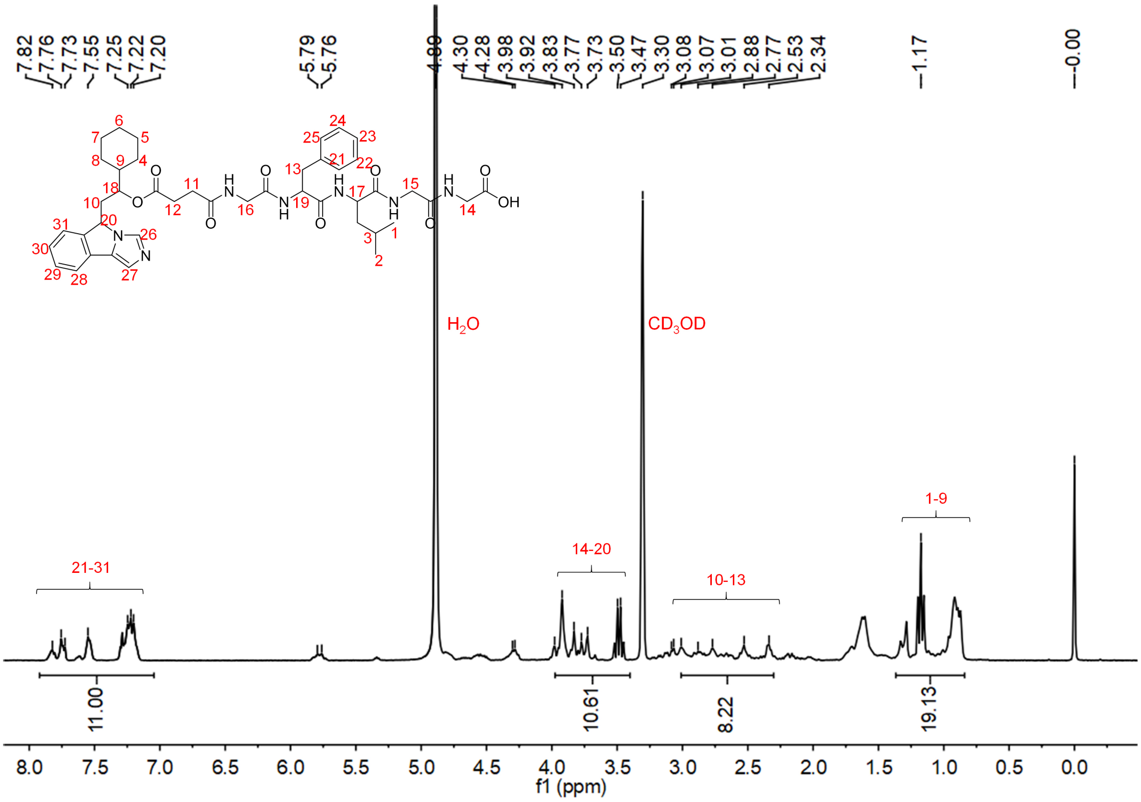

Supplementary Figure 8. ^{1}H NMR spectrum of the chimeric peptide ICP in CD_3OD.

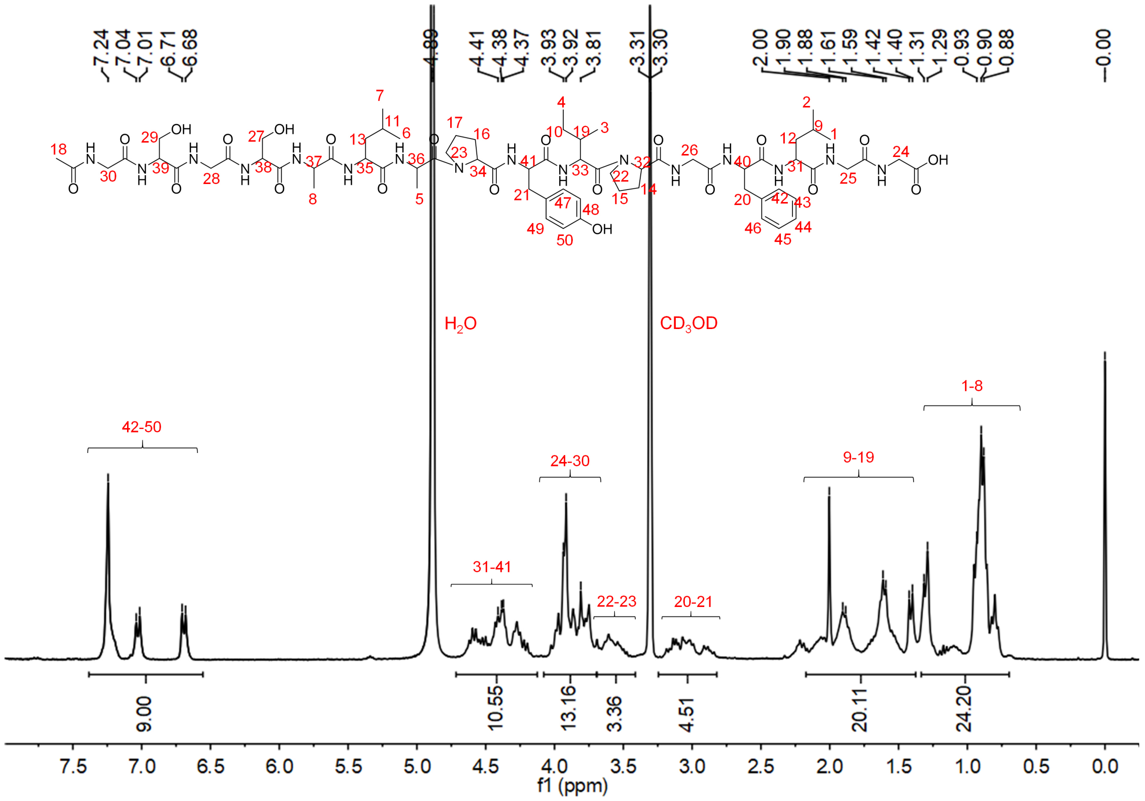

Supplementary Figure 9. ^{1}H NMR spectrum of the chimeric peptide PCP in CD_3OD.

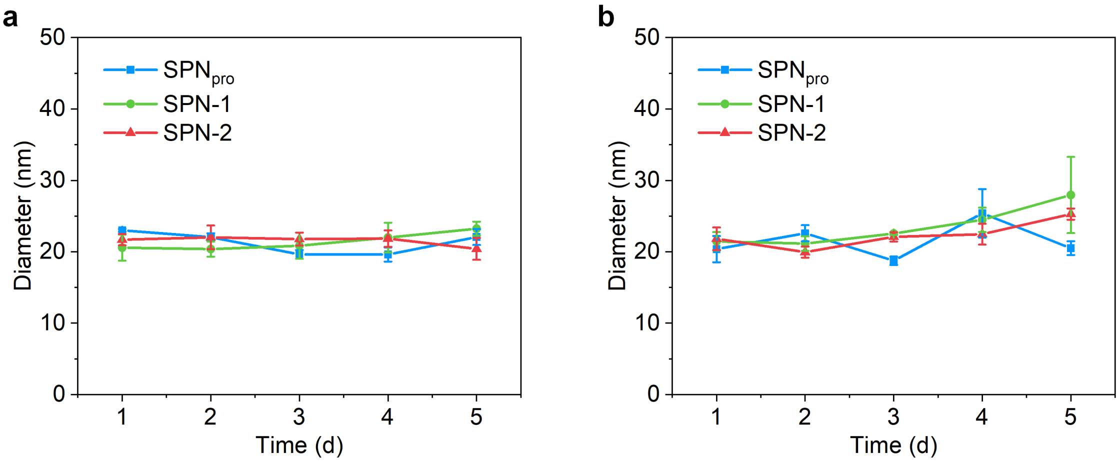

Supplementary Figure 10. DLS profiles of SPN_{pro}, SPN-1, and SPN-2 in 1× PBS buffer (pH 7.4) (a) and 10% fetal bovine serum (FBS) (b) solution for 5 days.

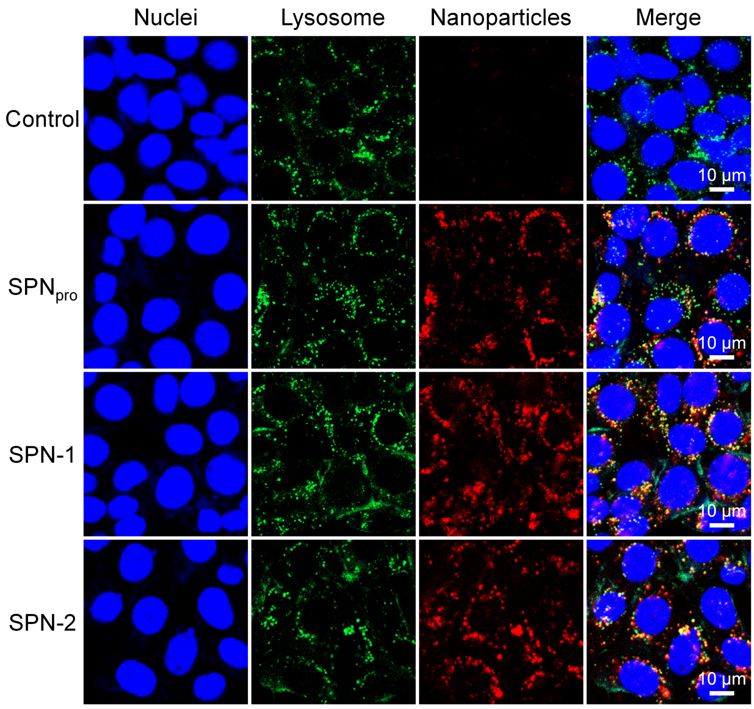

Supplementary Figure 11. Confocal fluorescence images of 4T1 cells after 12 h incubation with SPN_{pro}, SPN-1, or SPN-2 ([PCB]=20 μg/mL), followed by staining with lysosome tracker (Green DND-26) and cell nuclei dye (Hoechst 33342). The experiments were repeated independently three times with similar results.

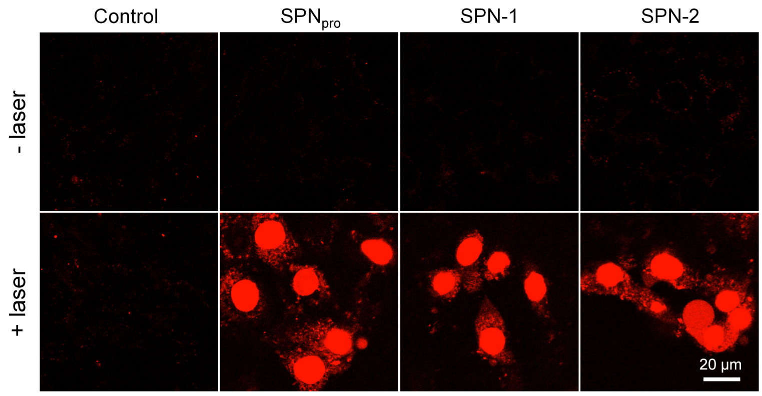

Supplementary Figure 12. *In vitro* $^1O_2$-mediated cytotoxicity analysis. Confocal fluorescence images of 4T1 cells after 12 h incubation with SPN_{pro}, SPN-1, and SPN-2 ([PCB]=20 μg/mL), followed by staining with PI with or without NIR photoirradiation (0.3 W/cm^2 at 808 nm) for 6 min. Red fluorescence indicated the signals from PI. The experiments were repeated independently three times with similar results.

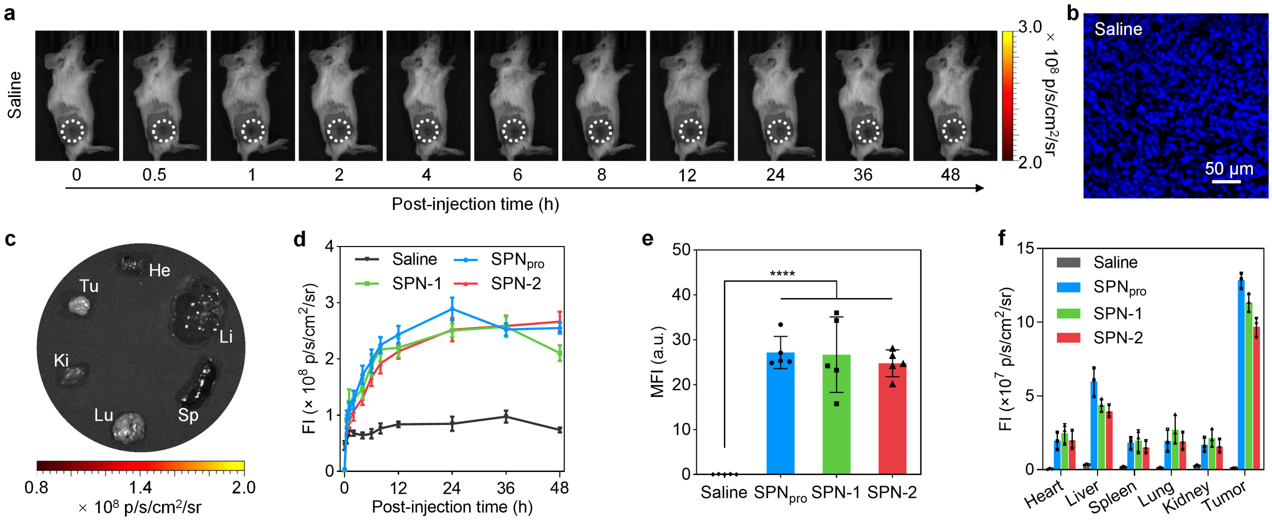

Supplementary Figure 13. *In vivo* tumor accumulation of SPNs. (a) NIR fluorescence imaging of 4T1 tumor-bearing BALB/c mice at different time points after the intravenous injection of saline (200 μL). (b) Confocal fluorescence images of primary tumors from 4T1 tumor-bearing mice after intravenous injection of saline. (c) *Ex vivo* NIR fluorescence images of tumors and major organs in 4T1 tumor-bearing mice at 48 h after intravenous injection of saline. (d) Quantitative NIR fluorescence intensity (FI) of primary tumors at different time points after the intravenous injection of saline, SPN_{pro}, SPN-1, or SPN-2 (n=3). (e) Quantitative analysis of the MFI in primary tumor tissues in 4T1 tumor-bearing mice at 48 h after intravenous injection of saline, SPN_{pro}, SPN-1, and SPN-2 (n=5). ****p<0.0001. (f) Quantitative NIR fluorescence intensity of tumors and major organs in 4T1 tumor-bearing mice at 48 h after intravenous injection of saline, SPN_{pro}, SPN-1, and SPN-2 (200 μL, [PCB]=200 μg/mL) (n=3). Statistical significance in (e) was calculated via one-way ANOVA with a Tukey posthoc test. ****p<0.0001. The mean values and SD are presented. The experiments in (b) were repeated independently three times with similar results.

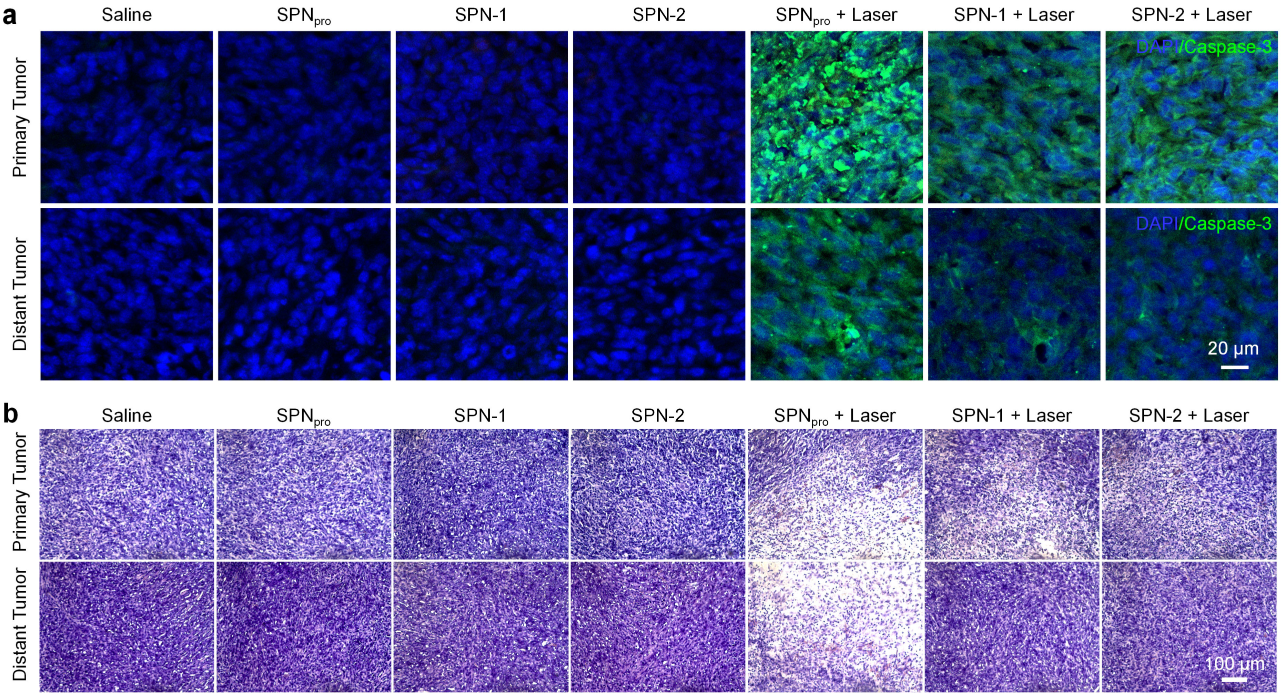

Supplementary Figure 14. *In vivo* SPNs-mediated antitumor therapy. (a) Immunofluorescence staining images of caspase-3 in primary and distant tumor tissues of 4T1 tumor-bearing mice after different treatments. The cell nucleus stained with DAPI and caspase-3 stained with antibodies showed blue and green fluorescence signals, respectively. (b) Histological H&E staining of primary and distant tumors in 4T1 tumor-bearing mice after different treatments. The experiments were repeated independently three times with similar results.

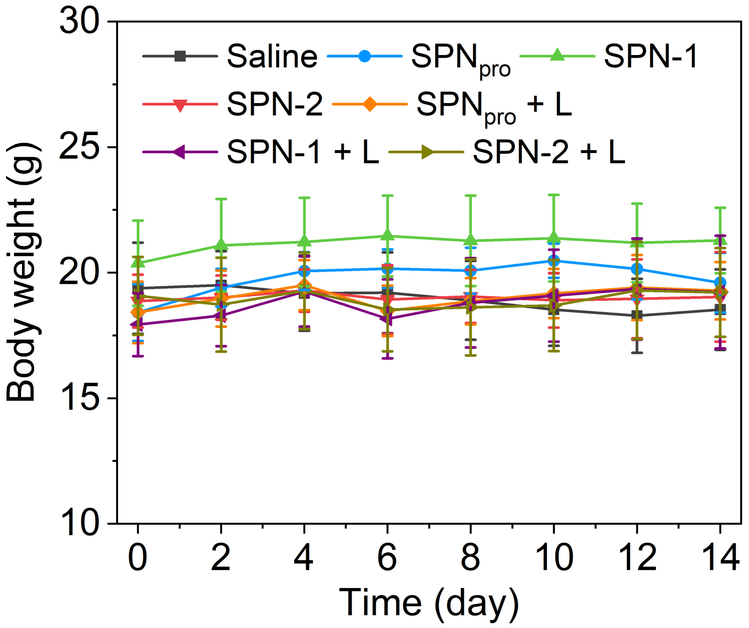

Supplementary Figure 15. Body weights of 4T1 tumor-bearing mice after different treatments. 4T1 tumor-bearing mice were intravenously injected with saline, SPN$_{pro}$, SPN-1, and SPN-2 (200 μL, [PCB]=200 μg/mL), and the primary tumors were treated with or without NIR photoirradiation (0.3 W/cm^2 at 808 nm) for 6 min (*n*=5).

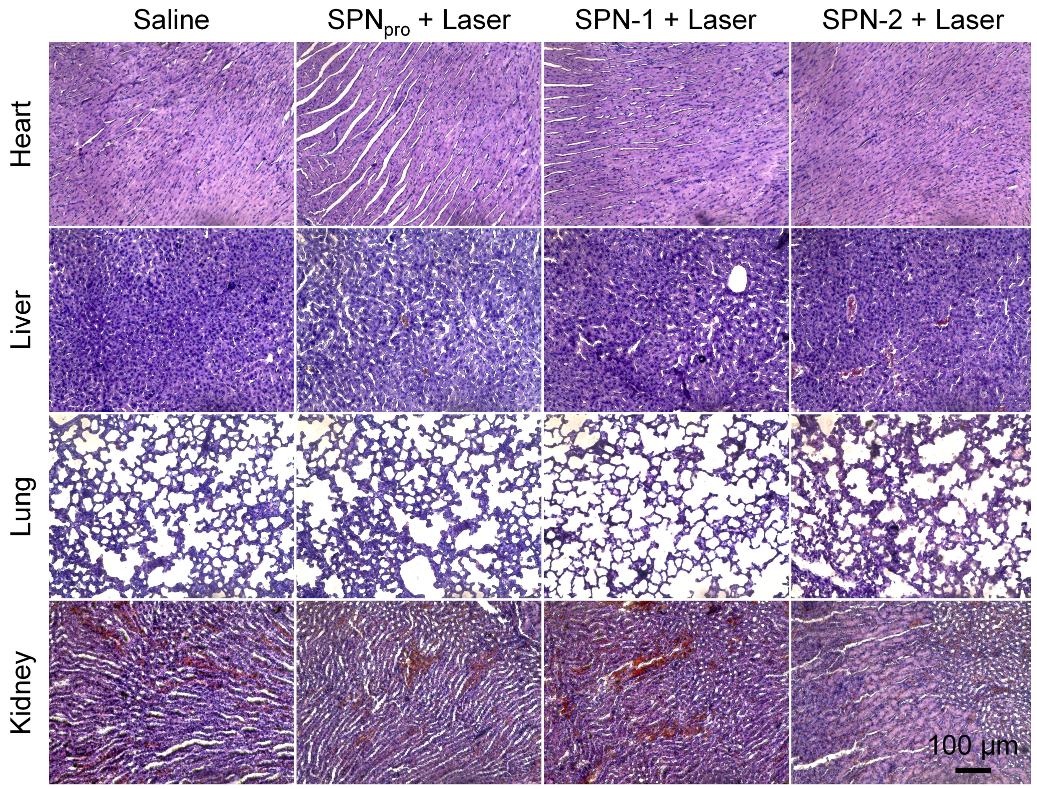

Supplementary Figure 16. Histological analysis of the major organs. Histological H&E staining of heart, liver, lung, and kidney from 4T1 tumor-bearing mice at day 14 after intravenous injection of saline, SPN_{pro}, SPN-1, and SPN-2 (200 μL, [PCB]=200 μg/mL) with or without NIR photoirradiation (0.3 W/cm^2 at 808 nm) for 6 min. The experiments were repeated independently three times with similar results.

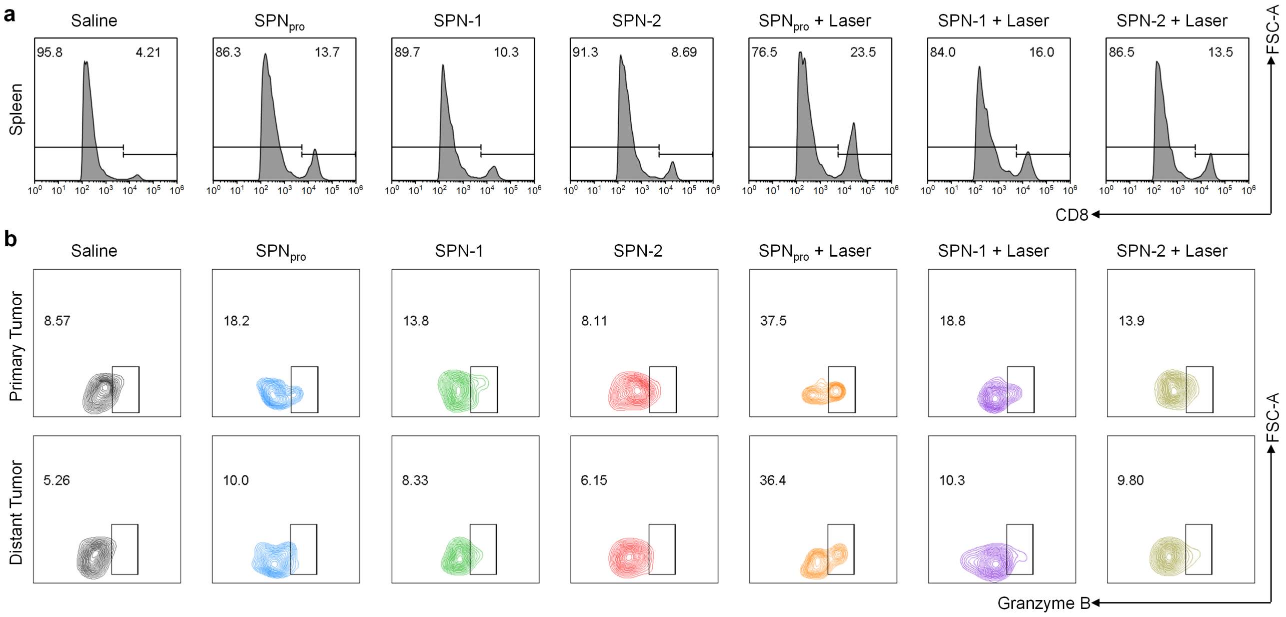

Supplementary Figure 17. FACS assay of splenic T lymphocytes ($CD3^+$ and $CD8^+$) (a) and granzyme B producing cytotoxic TILs ($CD8^+$ and granzyme B^+) (b) in 4T1 tumor-bearing mice after different treatments. 4T1 tumor-bearing mice were intravenously injected with saline, SPN_{pro}, SPN-1, and SPN-2 (200 μL, [PCB]=200 μg/mL), and the primary tumors were treated with or without NIR photoirradiation (0.3 W/cm^2 at 808 nm) for 6 min.

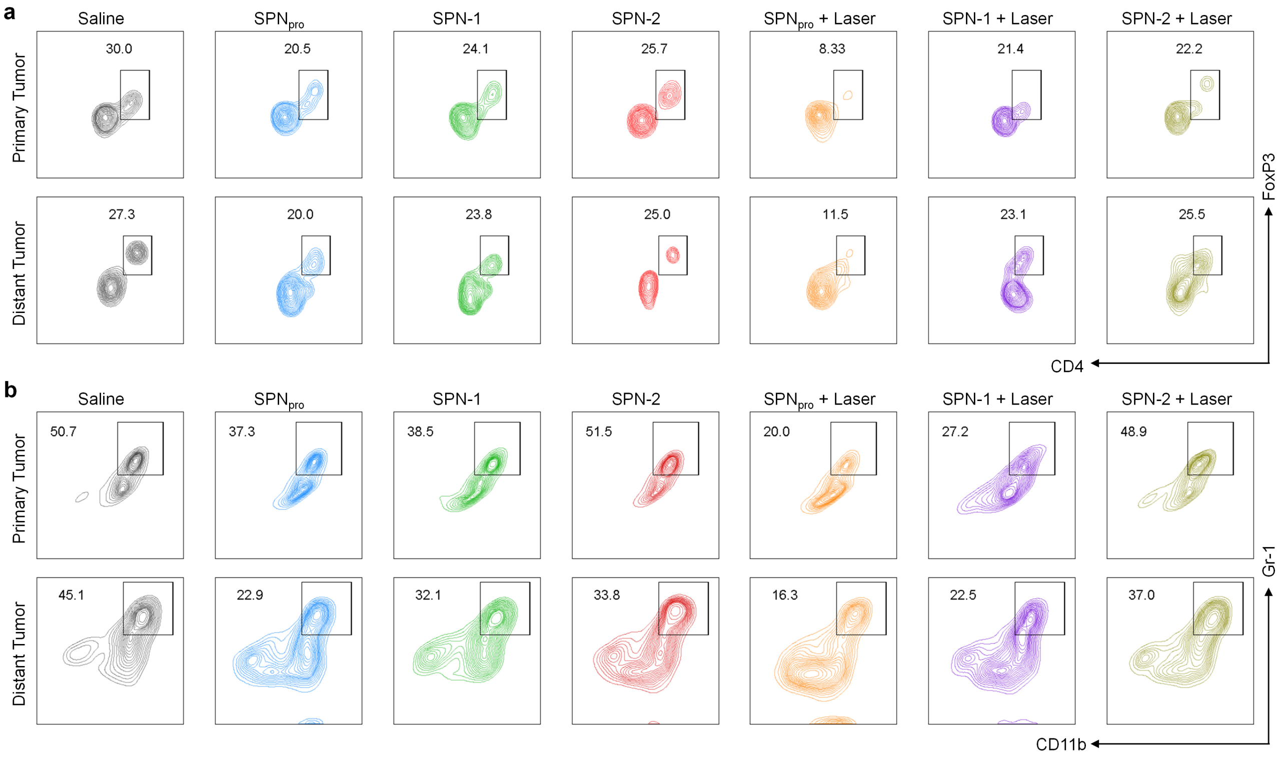

Supplementary Figure 18. FACS assay of regulatory T cells ($CD4^{+}Foxp3^{+}$) (a) and myeloid-derived suppressor cells (MDSCs) ($CD11b^{+}Gr\text{-}1^{+}$) (b) of the primary and distant tumors in 4T1 tumor-bearing mice after different treatments. 4T1 tumor-bearing mice were intravenously injected with saline, SPN_{pro}, SPN-1, and SPN-2 (200 μL, [PCB]=200 μg/mL), and the primary tumors were treated with or without NIR photoirradiation (0.3 W/cm^2 at 808 nm) for 6 min.

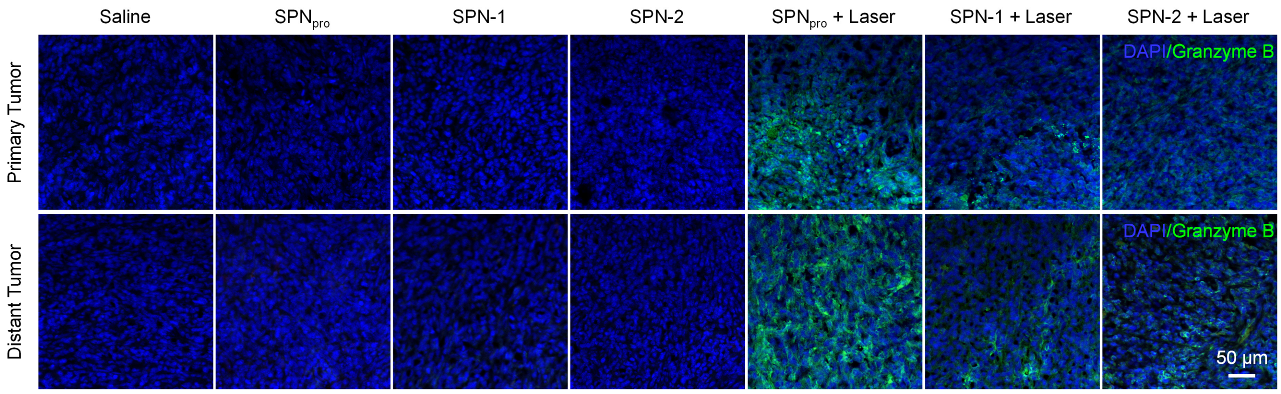

Supplementary Figure 19. Immunofluorescence staining images of granzyme B in primary and distant tumor tissues of 4T1 tumor-bearing mice after different treatments. The cell nucleus stained with DAPI and granzyme B stained with antibodies showed blue and green fluorescence signals,

respectively. 4T1 tumor-bearing mice were intravenously injected with saline, SPN_{pro}, SPN-1, and SPN-2 (200 μL, [PCB]=200 μg/mL), and the primary tumors were treated with or without NIR photoirradiation (0.3 W/cm^2 at 808 nm) for 6 min. The experiments were repeated independently three times with similar results.

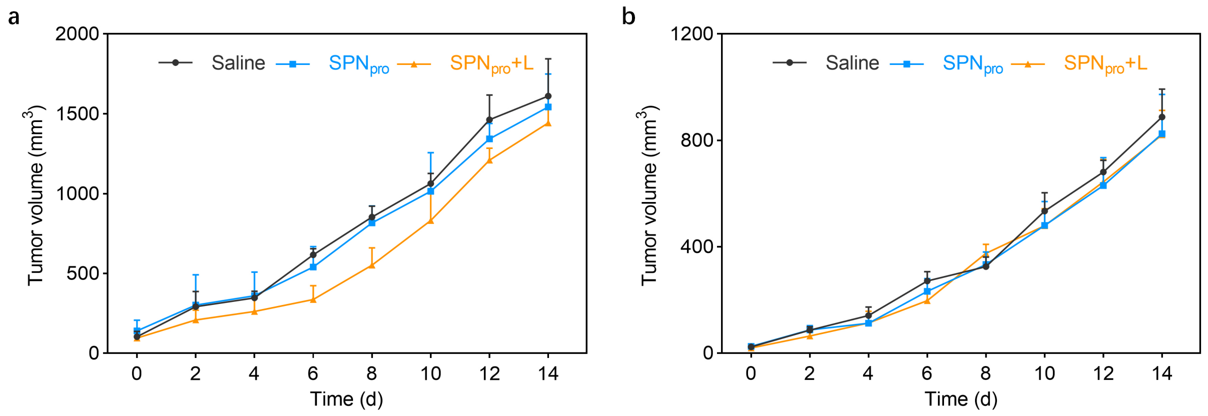

Supplementary Figure 20. Growth curves of primary tumors (a) and distant tumors (b) in 4T1 tumor-bearing NSG mice after different treatments (*n*=5). 4T1 tumor-bearing NSG mice were intravenously injected with saline and SPN_{pro} (200 μL, [PCB]=200 μg/mL), and the primary tumors were treated with or without NIR photoirradiation (0.3 W/cm^2 at 808 nm) for 6 min.

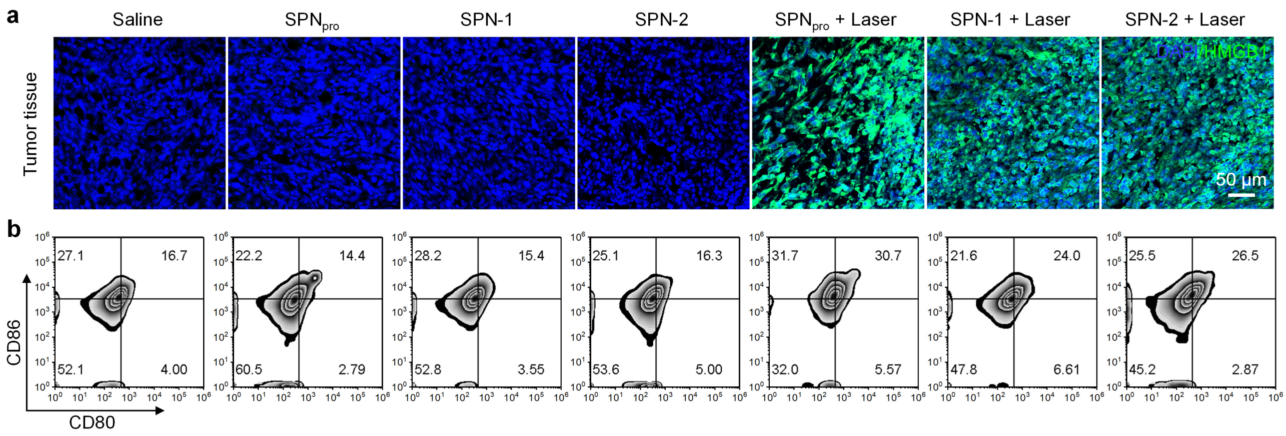

Supplementary Figure 21. (a) Confocal fluorescence images of primary tumors from 4T1 tumor-bearing mice after different treatments. The cell nucleus stained with DAPI and HMGB1 stained with antibodies showed blue and green fluorescence signals, respectively. (b) FACS assay of matured DCs ($CD80^+$ and $CD86^+$) from the tumor-draining lymph nodes in 4T1 tumor-bearing mice after different

treatments. 4T1 tumor-bearing mice were intravenously injected with saline, SPN_{pro}, SPN-1, and SPN-2 (200 μL, [PCB]=200 μg/mL), and the primary tumors were treated with or without NIR photoirradiation (0.3 W/cm^2 at 808 nm) for 6 min. The experiments in (a) were repeated independently three times with similar results.

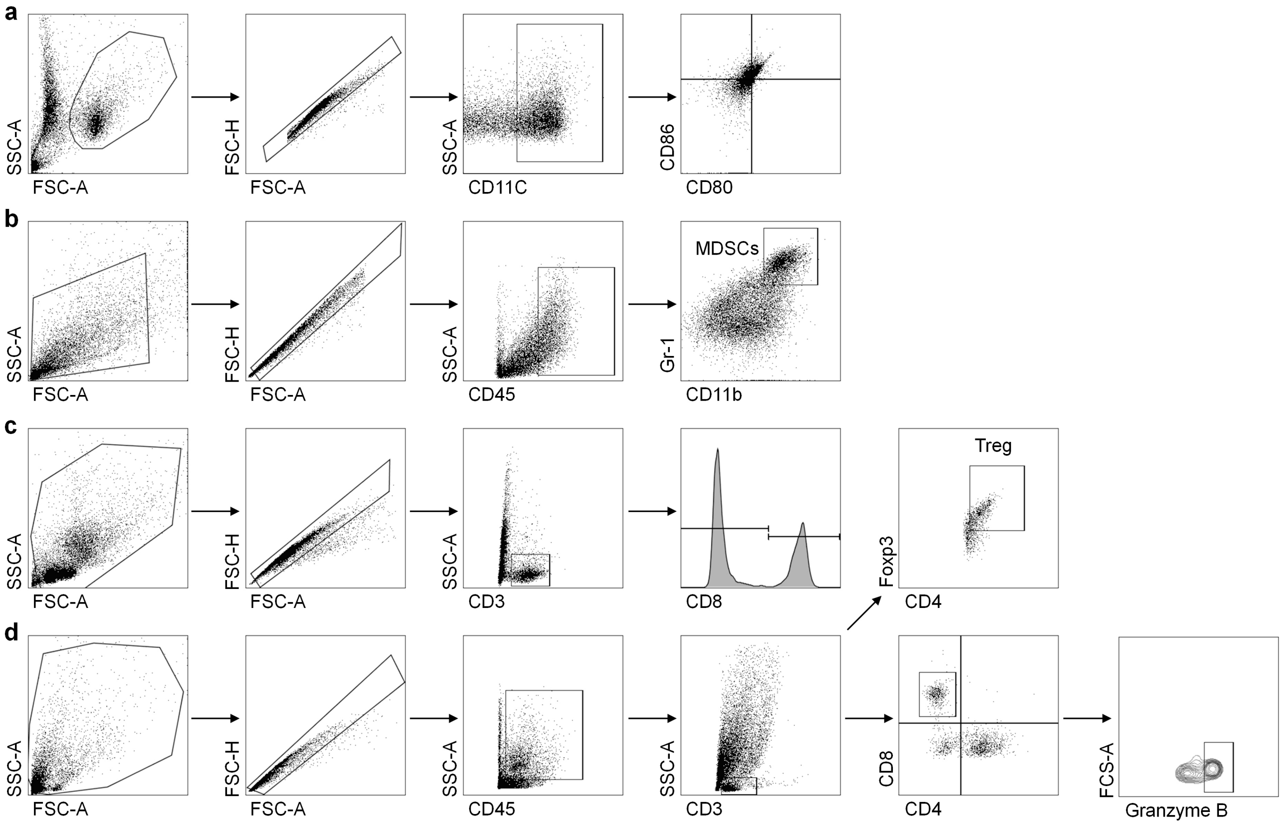

Supplementary Figure 22. Gating strategies used for flow cytometry analysis of immune cells. (a) Gating strategy to analyze matured DCs ($CD80^+$ and $CD86^+$) from the tumor-draining lymph nodes in 4T1 tumor-bearing mice. (b) Gating strategy to analyze MDSCs ($CD11b^+Gr\text{-}1^+$) from the tumors in 4T1 tumor-bearing mice. (c) Gating strategy to analyze splenic T lymphocytes ($CD3^+$ and $CD8^+$) from the spleens in 4T1 tumor-bearing mice. (d) Gating strategies to analyze regulatory T cells ($CD4^+Foxp3^+$), $CD8^+$ T cells, and granzyme B producing cytotoxic TILs ($CD8^+$ and granzyme B^+) from the tumors in 4T1 tumor-bearing mice.

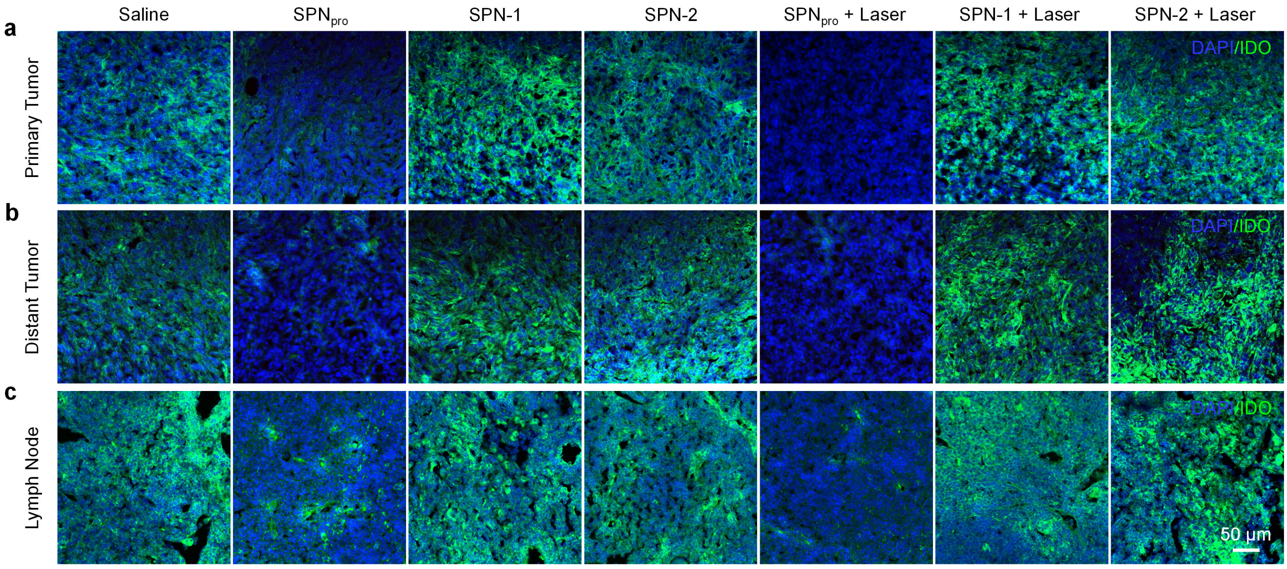

Supplementary Figure 23. *In vivo* SPN$_{pro}$-mediated IDO degradation. Immunofluorescence staining images of IDO in primary tumors (a), distant tumors (b), and tumor-draining lymph nodes (c) of 4T1 tumor-bearing mice after different treatments. The cell nucleus stained with DAPI and IDO stained with antibodies showed blue and green fluorescence signals, respectively. The experiments were repeated independently three times with similar results.
