# Supplementary material for: Semiconducting polymer nano-PROTACs for activatable photo-immunometabolic cancer therapy
Source: Nat Commun. 2021 May 18;12:2934. doi: 10.1038/s41467-021-23194-w (PMC8131624; doi:10.1038/s41467-021-23194-w)
Supplement: Supplementary file 2 — Reporting Summary [file 41467_2021_23194_MOESM2_ESM.pdf]

nature research

Corresponding author(s): Kanyi Pu
Last updated by author(s): Mar 26, 2021

Reporting Summary

Nature Research wishes to improve the reproducibility of the work that we publish. This form provides structure for consistency and transparency in reporting. For further information on Nature Research policies, see our Editorial Policies and the Editorial Policy Checklist.

Statistics

For all statistical analyses, confirm that the following items are present in the figure legend, table legend, main text, or Methods section.

n/a	Confirmed	
☐	☒	The exact sample size (*n*) for each experimental group/condition, given as a discrete number and unit of measurement
☐	☒	A statement on whether measurements were taken from distinct samples or whether the same sample was measured repeatedly
☐	☒	The statistical test(s) used AND whether they are one- or two-sided<br>*Only common tests should be described solely by name; describe more complex techniques in the Methods section.*
☐	☒	A description of all covariates tested
☐	☒	A description of any assumptions or corrections, such as tests of normality and adjustment for multiple comparisons
☐	☒	A full description of the statistical parameters including central tendency (e.g. means) or other basic estimates (e.g. regression coefficient) AND variation (e.g. standard deviation) or associated estimates of uncertainty (e.g. confidence intervals)
☐	☒	For null hypothesis testing, the test statistic (e.g. *F*, *t*, *r*) with confidence intervals, effect sizes, degrees of freedom and *P* value noted<br>*Give P values as exact values whenever suitable.*
☒	☐	For Bayesian analysis, information on the choice of priors and Markov chain Monte Carlo settings
☒	☐	For hierarchical and complex designs, identification of the appropriate level for tests and full reporting of outcomes
☒	☐	Estimates of effect sizes (e.g. Cohen's *d*, Pearson's *r*), indicating how they were calculated

Our web collection on statistics for biologists contains articles on many of the points above.

Software and code

Policy information about availability of computer code

Data collection	Irrelevant to experiments.
Data analysis	Fluorescence images were analyzed using the Living Image 4.3 Software (PerkinElmer) or ImageJ v1.51a Software. NMR spectra were analyzed using Mestre Nova LITE v5.2.5-4119 software (Mestre lab Research S.L.). Statistical calculations were performed using GraphPad Prism 8.0. Flow cytometry results were analyzed by FACS Diva and FlowJo v10.

For manuscripts utilizing custom algorithms or software that are central to the research but not yet described in published literature, software must be made available to editors and reviewers. We strongly encourage code deposition in a community repository (e.g. GitHub). See the Nature Research guidelines for submitting code & software for further information.

Data

Policy information about availability of data

All manuscripts must include a data availability statement. This statement should provide the following information, where applicable:
- Accession codes, unique identifiers, or web links for publicly available datasets
- A list of figures that have associated raw data
- A description of any restrictions on data availability

The authors declare that all related to this study are available in the article/and or its supplementary information files.

Field-specific reporting

Please select the one below that is the best fit for your research. If you are not sure, read the appropriate sections before making your selection.

- [x] Life sciences
- [ ] Behavioural & social sciences
- [ ] Ecological, evolutionary & environmental sciences

For a reference copy of the document with all sections, see nature.com/documents/nr-reporting-summary-flat.pdf

Life sciences study design

All studies must disclose on these points even when the disclosure is negative.

Sample size	We used G*power analysis to calculate and ensure the sample sizes fulfill adequate power (p>0.8). According to the experimental data and sample size (n), P value and effect size were calculated and the power was then calculated. If it is more than 80%, demonstrating the sample size is adequate.
Data exclusions	No data was excluded from this study.
Replication	Experiments were repeated at least three independent experiments with similar results. All experiments were reproduced to reliably support conclusions stated in the manuscript.
Randomization	Cages of mice were randomly selected and then divided into experimental groups for further treatment.
Blinding	Investigators were blinded to group allocation during experiments.

Reporting for specific materials, systems and methods

We require information from authors about some types of materials, experimental systems and methods used in many studies. Here, indicate whether each material, system or method listed is relevant to your study. If you are not sure if a list item applies to your research, read the appropriate section before selecting a response.

Materials & experimental systems

n/a	Involved in the study	
☐	☒	Antibodies
☐	☒	Eukaryotic cell lines
☒	☐	Palaeontology and archaeology
☐	☒	Animals and other organisms
☒	☐	Human research participants
☒	☐	Clinical data
☒	☐	Dual use research of concern

Methods

n/a	Involved in the study	
☒	☐	ChIP-seq
☐	☒	Flow cytometry
☒	☐	MRI-based neuroimaging

Antibodies

Antibodies used	PE-CD11b antibody (Catalog no. 101208), BV605-Gr-1 antibody (Catalog no. 108440), AF647-Foxp3 antibody (Catalog no. 126408), APC anti-mouse CD11c (Catalog no. 117310), FITC anti-mouse CD80 (Catalog no. 104706), PE anti-mouse CD86 (Catalog no. 105008), FITC anti-mouse CD3 (Catalog no. 100204), APC anti-mouse CD8 (Catalog no. 100712), PE anti-mouse CD4 (Catalog no. 130310), HMGB1 antibody (Catalog no. 651402) and purified anti-mouse CD16/32 (Catalog no. 156604) were purchased from Biolegend. IDO antibody (Catalog no. ab106134), granzyme B antibody (ab255598), and secondary antibody Alexa Fluor 488 conjugated goat anti-rabbit IgG H&L (ab150077) were purchased from Abcam. HMGB1 antibody (Catalog no. 3935S) and cleaved caspase-3 antibody (Catalog no. 9661L) were purchased from Cell Signaling Technology.
Validation	All antibodies were commercially available and were validated by the supplier. All antibodies were used in the study according to the profile of manufacturers.

Eukaryotic cell lines

Policy information about cell lines

Cell line source(s)	4T1 murine mammary carcinoma cell line were purchased from American Type Culture Collection, ATCC.
Authentication	These cell lines were authenticated by the supplier using STR analysis.
Mycoplasma contamination	No contamination was detected by the supplier using Hoechst DNA stain method, agar culture method, and PCR-based assay.

Commonly misidentified lines (See ICLAC register)	These cell lines that we used were not listed in commonly misidentified lines in ICLAC Register.

Animals and other organisms

Policy information about studies involving animals; ARRIVE guidelines recommended for reporting animal research

Laboratory animals	Female BALB/c and NSG mice (6 weeks old) were purchased from InVivos Pte Ltd (Singapore).
Wild animals	No wild animals were used in this study.
Field-collected samples	No field collected samples were used in this study.
Ethics oversight	Animal experiments in Singapore were performed in compliance with Guidelines for Care and Use of Laboratory Animals of the Nanyang Technological University-Institutional Animal Care and Use Committee (NTU-IACUC) and approved by the Institutional Animal Care and Use Committee (IACUC) for Animal Experiment, Singapore.

Note that full information on the approval of the study protocol must also be provided in the manuscript.

Flow Cytometry

Plots

Confirm that:

- [ ] The axis labels state the marker and fluorochrome used (e.g. CD4-FITC).
- [ ] The axis scales are clearly visible. Include numbers along axes only for bottom left plot of group (a 'group' is an analysis of identical markers).
- [ ] All plots are contour plots with outliers or pseudocolor plots.
- [x] A numerical value for number of cells or percentage (with statistics) is provided.

Methodology

Sample preparation	Tumor samples were digested with 1 mg/mL type I collagenase, 100 µg/mL type IV collagenase, and 100 µg/ml DNase I for 2 h at 37 °C, then the tissues were filtered through a 70 µm nylon cell strainer. Then the red blood cells of the obtained tumor single cell suspension were removed using ACK lysis, and the cells were washed thrice with cell staining buffer. Single-cell suspensions were obtained and stained with antibodies according to the manufacturer's protocols, and then analyzed by flow cytometry.
Instrument	Fortessa X20 (BD Biosciences)
Software	FACS Diva and FlowJo v10
Cell population abundance	No cell sorting was performed.
Gating strategy	In general, cells were first gated on FSC/SSC. Singlet cells were gated using FSC-H and FSC-A. Dead cells were then excluded and further surface and intracellular antigen gating was performed on the live cell population.

- [x] Tick this box to confirm that a figure exemplifying the gating strategy is provided in the Supplementary Information.
